# *Reluctance to Downplay: Asymmetric Sensitivity to Differences in the Severity of Moral Transgressions

**DOI:** 10.1177/09567976251314972

**Published:** 2025-03-19

**Authors:** Amanda E. Geiser, Ike Silver, Deborah A. Small

**Affiliations:** 1 Haas School of Business, University of California, Berkeley; 2 Marshall School of Business, University of Southern California; 3 Yale School of Management, Yale University

**Keywords:** moral judgment, condemnation, punishment, comparison, order effects, framing, open data, open materials, preregistered

## Abstract

A common-sense moral intuition is that bad acts should be condemned according to severity. Yet seven experiments (*N* = 6,075 U.S. adults) show that the extent to which people differentiate between transgressions hinges on the direction of comparison. When scaling up from a less severe transgression to a more severe one, people readily express stronger condemnation of the worse transgression. But when scaling down from a more severe transgression to a less severe one, they differentiate less, often condemning the lesser transgression just as strongly as one that is transparently worse. Indicating that one transgression is less bad than another can be construed as downplaying such transgressions, signaling bad moral character. Supporting this account, the asymmetry is larger for judgments that implicate moral character and for transgressions that seem especially important to condemn. Observers’ moral-character judgments reveal a similar pattern, suggesting that the asymmetry is reinforced by social incentives.

People often decide how harshly to condemn a bad act by comparing it to other bad acts. Consider, for example, a public figure accused of harassment by his employees. Without a point of comparison, it can be hard to judge how much punishment such behavior deserves. For instance, observers may not know how much weight to give to the precise number of victims or the amount of harm they suffered. But when comparing misconduct cases, observers can more easily assess their relative severity. As in other domains, comparing cases highlights relevant differences between them, facilitating sensitivity to the scope of each case (Hsee, 1996; Hsee et al., 1999, 2005). Drawing comparisons can thus help observers differentiate between bad acts and ensure that each is punished in proportion to its severity (Kahneman et al., 1998, 1999; Sunstein et al., 2001).

In this research, we propose that the extent to which people differentiate between transgressions hinges on a seemingly irrelevant factor: the direction of comparison. When comparing transgressions that differ in severity (e.g., in the number of victims or the degree of harm), one can either start with the less severe case and scale up to the more severe case or start with the more severe case and scale down to the less severe case. Although the direction of comparison ought not influence the relative amount of condemnation each transgression receives, we find that people adjust condemnation asymmetrically depending on whether the context requires upward or downward comparison. People readily differentiate between transgressions when scaling up, but they differentiate between the same transgressions much less—and often not at all—when scaling down.

We propose that this asymmetry arises because people are reluctant to downplay bad acts—that is, they are motivated to react with sufficient concern. One’s willingness to condemn transgressions is seen as a signal of moral character: Good people are expected to punish bad acts (Barclay, 2006; Boyd et al., 2003; Fehr & Gächter, 2002). Supporting this idea, evidence from scenario studies and economic games reveals that third parties who punish moral transgressions are socially rewarded (Jordan & Kteily, 2023; Jordan & Rand, 2017; Jordan et al., 2016), whereas those who fail to punish incur reputational costs (Boyd & Richerson, 1992; Martin et al., 2019). Because people understand that the extent to which they condemn bad acts implicates their moral character, they are reluctant to express insufficient condemnation.

When comparing transgressions that differ in severity, people may feel as though they are downplaying to different degrees depending on the direction of comparison. Scaling down from a more severe transgression to a less severe one requires people to indicate that one case is less bad than another, which may suggest to others that they believe such transgressions are not very bad (Inbar & Evers, 2022). By contrast, when scaling up, differentiating between transgressions creates no such ambiguity: Indicating that one case is worse than another does not risk downplaying either case. Therefore, people may resist differentiating between transgressions when scaling down just as they reject other comparisons that threaten their morals (Baron & Spranca, 1997; Tetlock et al., 2000), despite being perfectly willing to differentiate when scaling up.

In sum, we predict a directional asymmetry in people’s willingness to differentiate between bad acts: To avoid expressing insufficient condemnation, people scale down condemnation less than they scale up. In line with this account, we expect the asymmetry to be more pronounced for judgments that implicate moral character to a greater extent and for transgressions that seem especially important to condemn. Moreover, we expect a similar asymmetry to emerge in observers’ judgments of the morality of scaling up versus scaling down.

**Statement of Relevance**

People often decide how harshly to condemn a transgression by comparing it with other similar transgressions. For example, after dozens of women accused Harvey Weinstein of sexual abuse in 2017, others came forward with similar allegations against other celebrities, offering a frame of reference that helped observers discern just how bad each case was. This research shows that the extent to which comparison aids in differentiating between transgressions hinges on its direction. People readily differentiate when starting with a less severe transgression and scaling up to a worse one, but they differentiate between the same transgressions much less—and often not at all—when scaling down. Saying that one transgression is less bad than another can be construed as insufficiently condemning such transgressions (i.e., downplaying them), thus raising doubts about one’s moral character. We discuss how this psychology may contribute to broader trends in how the public responds to and punishes wrongdoings.

## Open Practices Statement

We report nine studies (seven in the main manuscript and two in Supplemental Material; total *N* = 7,455). All studies were preregistered on AsPredicted. Preregistrations, materials, data, and code are available at https://researchbox.org/928&PEER_REVIEW_passcode=ANAFYW. The target sample sizes for all studies were specified in advance in our preregistrations, as were all exclusion criteria, manipulations, measures, and analyses. Generally, we aimed to recruit at least 200 participants per condition to ensure adequate statistical power. Additional analyses and minor deviations from our preregistrations are detailed in the Supplemental Material available online.

## Research Transparency Statement

### General disclosures

All preregistrations, materials, data, and code are publicly available and posted on ResearchBox (https://researchbox.org/928&PEER_REVIEW_passcode=ANAFYW). **Conflicts of interest:** The authors declare no conflicts of interest. **Funding:** This research was supported by funding from the Wharton School of the University of Pennsylvania and Yale School of Management. **Artificial intelligence:** No artificial-intelligence-assisted technologies were used in this research or the creation of this article. **Ethics:** This research received approval from the institutional review boards of the University of Pennsylvania and Yale University.

### Study 1 disclosures

**Preregistration:** The research question, manipulations, measures, analyses, sample size, and exclusion criteria were preregistered on AsPredicted before data collection began. There were no major deviations from the preregistration. However, there was a minor typo in the description of the punishment measure in the preregistration (for further details, see page 43 of the Supplemental Material). **Materials:** Study materials are publicly available. **Data:** Raw data files are publicly available. **Analysis scripts:** Analysis scripts are publicly available.

### Studies 2a and 2b disclosures

**Preregistration:** The research question, manipulations, measures, analyses, sample size, and exclusion criteria were preregistered on AsPredicted before data collection began. There were no major deviations from the preregistration. However, the coding of the binary dependent variable deviated slightly from the preregistered plan; rather than coding those who did differentiate between cases as 1 and those who did not differentiate as 0, as preregistered, we coded those who did not differentiate as 1 and those who did differentiate as 0. **Materials:** Study materials are publicly available. **Data:** Raw data files are publicly available. **Analysis scripts:** Analysis scripts are publicly available.

### Study 3 disclosures

**Preregistration:** The research question, manipulations, measures, analyses, sample size, and exclusion criteria were preregistered on AsPredicted before data collection began. There were no major or minor deviations from the preregistration. **Materials:** Study materials are publicly available. **Data:** Raw data files are publicly available. **Analysis scripts:** Analysis scripts are publicly available.

### Study 4 disclosures

**Preregistration:** The research question, manipulations, measures, analyses, sample size, and exclusion criteria were preregistered on AsPredicted before data collection began. There were no major or minor deviations from the preregistration. **Materials:** Study materials are publicly available. **Data:** Raw data files are publicly available. **Analysis scripts:** Analysis scripts are publicly available.

### Study 5 disclosures

**Preregistration:** The research question, manipulations, measures, analyses, sample size, and exclusion criteria were preregistered on AsPredicted before data collection began. There was one deviation from the preregistered analysis plan: Our preregistration stated that we would test for the interaction between comparison frame and each potential moderator of the asymmetry using logistic mixed-effects regressions. However, because these models failed to converge, we instead used linear mixed-effects regressions. Our preregistered logistic regression models, despite convergence issues, yielded similar results. We include both sets of models in the code files posted on ResearchBox. **Materials:** Study materials are publicly available. **Data:** Raw data files are publicly available. **Analysis scripts:** Analysis scripts are publicly available.

### Study 6 disclosures

**Preregistration:** The research question, manipulations, measures, analyses, sample size, and exclusion criteria were preregistered on AsPredicted before data collection began. There were no major or minor deviations from the preregistration. **Materials:** Study materials are publicly available. **Data:** Raw data files are publicly available. **Analysis scripts:** Analysis scripts are publicly available.

## Study 1

Participants evaluated two real-world cases of misconduct in randomized order. We expected that they would adjust condemnation more when starting with the less severe case (scaling up) than when starting with the more severe case (scaling down).

### Method

#### Participants and design

We requested 1,200 participants from Prolific and received 1,202 complete submissions. Consistent with our preregistration, we excluded all submissions from participants who opened the survey more than once under the same participant ID or IP address (*n* = 34). The final sample consisted of 1,168 participants (49.2% men, 49.4% women, 1.4% other identity; mean age = 38.7 years). Participants were randomly assigned to one of two order conditions: the less-severe-first condition or the more-severe-first condition.

#### Procedure

Participants sequentially evaluated the sexual-misconduct cases of Harvey Weinstein, a film producer accused of violent sexual assault by more than 80 women, and Louis CK, a comedian accused of milder forms of sexual misconduct by five women. We selected these cases because although both are clearly wrong, they clearly differ in severity, both in the number of victims and in the severity of the targets’ actions. Participants in the less-severe-first condition considered Louis CK’s case first (and thus would have to adjust condemnation upward to indicate that Harvey Weinstein’s actions were worse), whereas those in the more-severe-first condition considered Harvey Weinstein’s case first (and thus would have to adjust condemnation downward to indicate that Louis CK’s actions were less bad).

At the start of the survey, participants were told that they would be asked to make judgments about celebrities accused of sexual misconduct. They were not explicitly told how many cases they would evaluate, and they were not told any specific details of either case. Participants read about and evaluated the first case on one page before proceeding to read about and evaluate the second case on a separate page. Each case was described in a short newslike blurb accompanied by a screenshot of a headline and image of the target that had appeared in *The New York Times* when the allegations surfaced. Neither blurb made reference to the other case.

For each case, participants judged how much the target should be punished on a scale from 0 (*no punishment*) to 10 (*the death penalty*). Next, they reported how morally outraged they were by the target’s actions on a scale from 0 (*not at all outraged*) to 10 (*the most outraged I could possibly be*). Finally, for exploratory purposes, we asked them to indicate whether they endorsed banning the target from the entertainment industry for an unspecified period of time (*yes* or *no*). All measures appeared on the same page for a given case.

### Results

To assess the amount of adjustment on each measure, we created two difference scores for each participant—one for the amount that they adjusted punishment between the two cases, and another for the amount that they adjusted moral outrage between the two cases. Adjustment of punishment was calculated by subtracting the amount of punishment assigned to Louis CK from the amount of punishment assigned to Harvey Weinstein, and adjustment of moral outrage was calculated by subtracting the amount of outrage expressed toward Louis CK from the amount of outrage expressed toward Harvey Weinstein. Larger difference scores thus reflect greater upward adjustment among those who considered Louis CK first and greater downward adjustment among those who considered Harvey Weinstein first.

#### Punishment

**Fig. 1.**
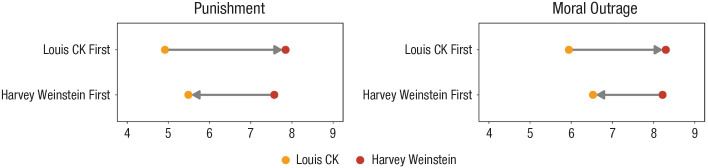
Adjustment of condemnation between Louis CK (less severe case) and Harvey Weinstein (more severe case) as a function of order in Study 1. Right-pointing arrows are used to indicate upward adjustment from Louis CK to Harvey Weinstein, and left-pointing arrows are used to indicate downward adjustment from Harvey Weinstein to Louis CK. The left side shows results for punishment (0 = *no punishment*, 10 = *the death penalty*), and the right side shows results for moral outrage (0 = *not at all outraged*, 10 = *the most outraged I could possibly be*). On both measures, participants made larger upward than downward adjustments, consistent with our predicted asymmetry.

We conducted a linear regression with the amount of adjustment in punishment predicted by order (−0.5 = less severe first, +0.5 = more severe first). In line with our predicted order-based asymmetry, participants adjusted punishment to a lesser extent if they evaluated Harvey Weinstein before Louis CK (*M* = 2.09, *SD* = 1.85) than if they evaluated Louis CK before Harvey Weinstein (*M =* 2.93, *SD* = 1.90), *t*(1166) = −7.71, *p* < .001. Mean punishment ratings for each case and adjustment between cases are displayed on the left side of Figure 1.

In addition to the amount of adjustment, we also explored how often participants adjusted punishment at all. Although the majority of participants in both order conditions adjusted by some amount, those who started with Harvey Weinstein refrained from differentiating (i.e., assigned equal punishment to both cases) more frequently (16.8%) than those who started with Louis CK (5.8%), χ^2^(1, *N* = 1,168) = 34.74, *p* < .001.

When examining the punishments assigned to each individual case, we found that both cases were influenced by order. Participants assigned more punishment to Harvey Weinstein if they evaluated his case after Louis CK’s (*M* = 7.85, *SD* = 1.36) than if they evaluated his case first (*M* = 7.57, *SD* = 1.45), *t*(1166) = −3.34, *p* < .001, and they assigned more punishment to Louis CK if they evaluated his case after Harvey Weinstein’s (*M* = 5.49, *SD* = 2.09) than if they evaluated his case first (*M* = 4.92, *SD* = 2.03), *t*(1166) = 4.73, *p* < .001.

#### Moral outrage

Next, we examined whether order influenced the amount of adjustment in moral outrage from one case to the next. As in punishment, participants adjusted moral outrage to a lesser extent from Harvey Weinstein to Louis CK (*M* = 1.69, *SD* = 1.78) than from Louis CK to Harvey Weinstein (*M =* 2.35, *SD* = 2.07), *t*(1166) = −5.86, *p* < .001. Those who evaluated Harvey Weinstein first were also more likely to refrain from differentiating at all (25.6%) than those who evaluated Louis CK first (12.2%), χ^2^(1, *N* = 1,168) = 34.50, *p* < .001. Mean ratings of moral outrage for each case and adjustment between cases are displayed on the right side of Figure 1.

Whereas order effects emerged for punishment of both Harvey Weinstein and Louis CK, order only influenced moral outrage toward Louis CK (the less severe case). Participants expressed no more outrage toward Harvey Weinstein if they considered his case after Louis CK’s (*M* = 8.30, *SD* = 1.73) than if they considered his case first (*M* = 8.22, *SD* = 1.75), *t*(1166) = 0.76, *p* = .445. But they expressed more outrage toward Louis CK if they considered his case after Harvey Weinstein’s (*M* = 6.53, *SD* = 2.37) than if they considered his case first (*M* = 5.94, *SD* = 2.42), *t*(1166) = 4.17, *p* < .001.

#### Endorsement of an entertainment industry ban

Finally, we explored how order of evaluation influenced support for banning each target from the entertainment industry. Nearly all participants agreed that Harvey Weinstein should be banned from the entertainment industry, regardless of whether they evaluated his case first (95.9%) or second (96.4%), χ^2^(1, *N* = 1,168) = 0.20, *p* = .657. When considering whether Louis CK deserved the same punishment, 64.2% of participants who evaluated his case first endorsed banning him from the entertainment industry. However, among those who evaluated Louis CK after Harvey Weinstein, the proportion endorsing such a ban increased to 70.4%, χ^2^(1, *N* = 1,168) = 5.23, *p* = .022. When examining how often participants differentiated between cases on this measure, we found that those who evaluated Harvey Weinstein first were more likely to respond in the same way to both cases (74.2%) than those who evaluated Louis CK first (67.4%), χ^2^(1, *N* = 1,168) = 6.49, *p* = .010.

## Studies 2a and 2b

Studies 2a and 2b conceptually replicated the order-based asymmetry observed in Study 1 using controlled pairs of transgressions that differed in severity quantitatively (Study 2a) and qualitatively (Study 2b).

### Method

#### Participants and design

We requested 600 participants from Prolific for each study. We received 601 complete submissions for Study 2a and 600 complete submissions for Study 2b. Consistent with our preregistrations, we excluded all submissions from participants who opened the survey more than once under the same participant ID or IP address (*n* = 16 in Study 2a; *n* = 16 in Study 2b). The final sample for Study 2a consisted of 585 participants (16.6% men, 81.5% women, 1.9% other identity; mean age = 26.9 years),^1^ and the final sample for Study 2b consisted of 584 participants (48.8% men, 49.0% women, 2.2% other identity; mean age = 39.6 years). Participants in each study were randomly assigned to one of two order conditions: the less-severe-first condition or the more-severe-first condition.

#### Procedure

Studies 2a and 2b followed the same procedure. Participants sequentially evaluated three pairs of transgressions (six cases in total): one pair of sexual-assault cases, one pair of robbery cases, and one pair of aggravated-assault cases. Within each pair, one case was transparently more severe than the other, but both cases explicitly stated that the target had been found guilty of the relevant crime. In Study 2a, each pair of transgressions included two cases that differed in severity quantitatively: These included a pair of sexual-assault cases (sexual assault of two vs. three children), a pair of robbery cases (robbing one vs. several convenience stores while armed with a gun), and a pair of aggravated-assault cases (attacking one person vs. two people with a knife). In Study 2b, each pair included two cases that differed qualitatively: These included a pair of sexual-assault cases (sexual assault involving an adult victim vs. a child victim), a pair of robbery cases (raising a fist at a cashier and threatening to hurt them vs. pointing a gun at a cashier and threatening to kill them), and a pair of aggravated-assault cases (attacking someone with a knife and inflicting cuts on their hands and arms vs. stab wounds on their chest and abdomen). The two cases within a given pair were always presented one after another (one case per page). Participants in the less-severe-first condition evaluated the less severe case first within each pair, and those in the more-severe-first condition evaluated the more severe case first within each pair. Transgression type was blocked and presented in random order.

For each case, participants first assigned a punishment length: “What do you think is an appropriate prison sentence for this offense? ___ years.” They entered responses into an open-ended text box. We required participants to respond with an integer value greater than zero, given that the description of each case specified that the target had been found guilty. Requiring responses to be greater than zero also ensured that the ratio between prison sentences (one of our primary dependent measures) would not be undefined. Next, participants rated how much punishment each case deserved using two scale-based measures similar to the punishment measure used in Study 1: “How much should [Target] be punished?” (1 = *no punishment*, 11 = *most extreme punishment allowable by law*) and “How severe a punishment should be given for this offense?” (1 = *not at all*, 11 = *extremely*). Responses to these two items (Study 2a: *r* = .94; Study 2b: *r* = .95) were averaged to create a composite.

Both studies also included two sets of exploratory measures. On the same page as the punishment measures, participants indicated how outraged they were about the case: “How morally outraged were you by this offense?” (1 = *not at all*, 11 = *extremely*). On a separate page at the end of the survey, they rated their moral conviction (Skitka & Morgan, 2014) for each type of transgression (e.g., aggravated assault), without reference to the more severe or less severe cases they saw earlier. For each transgression type, participants indicated to what extent their position is “a reflection of your core moral beliefs and convictions” and “connected to your beliefs about fundamental right and wrong” (1 = *not at all*, 5 = *very much*). Analyses of these measures are reported in the Supplemental Material, and they are not discussed further in the main manuscript.

### Results

To assess the extent to which participants differentiated between the less severe and more severe cases within each pair, we created a within-participant difference score for each participant and each punishment measure by subtracting the participant’s judgment of the less severe case from their judgment of the more severe case, following the same procedure as in Study 1. For the open-ended punishment-length measure (i.e., the number of years in prison), we also examined the ratio between punishment lengths assigned to each case by dividing the participant’s judgment of the more severe case by their judgment of the less severe case. Table 1 summarizes all indicators of punishment-length adjustment between cases for each pair of transgressions in each study.

**Table 1.** Mean Punishment Length for Each Case and Adjustment Between Cases in Studies 2a and 2b

		Sexual assault		Robbery		Aggravated assault	
Study	Measure	Less severe first	More severe first	Less severe first	More severe first	Less severe first	More severe first
Study 2a	Less severe case	30.39 (25.45)	38.40*** (29.69)	9.84 (9.06)	10.60 (9.99)	22.35 (20.47)	24.56 (20.65)
	More severe case	38.24 (29.07)	40.87 (29.82)	14.42 (13.67)	11.94* (10.80)	29.55 (25.4)	27.74 (22.18)
	Absolute adjustment	7.85 (9.90)	2.47*** (5.67)	4.59 (6.46)	1.34*** (3.78)	7.20 (10.57)	3.18*** (6.85)
	Ratio-based adjustment	1.34 (0.37)	1.10*** (0.20)	1.52 (0.53)	1.22*** (0.56)	1.39 (0.44)	1.18*** (0.31)
	Frequency of adjustment	76.0%	33.8%***	80.5%	33.8%***	73.3%	43.7%***
Study 2b	Less severe case	15.75 (12.89)	21.53*** (19.40)	6.40 (9.18)	6.80 (7.21)	12.31 (10.55)	15.45** (13.08)
	More severe case	25.75 (18.65)	23.40 (19.38)	12.33 (11.74)	11.85 (11.46)	19.00 (14.28)	20.72 (15.07)
	Absolute adjustment	9.99 (11.53)	1.87*** (5.07)	5.93 (9.91)	5.05 (8.03)	6.69 (7.69)	5.27* (7.67)
	Ratio-based adjustment	2.69 (11.71)	1.69 (7.83)	2.59 (2.01)	2.32 (2.97)	1.80 (0.97)	1.91 (5.81)
	Frequency of adjustment	82.1%	36.9%***	89.0%	76.1%***	83.9%	66.9%***

Note: Standard deviations are given in parentheses. Indications of statistical significance are based on linear or logistic regressions testing for order effects on each measure. *p < .05. **p < .01. ***p < .001.

**Table 2.** Size of the *More*/*Less* Asymmetry and Ratings of Moral-Signaling Relevance, Harmfulness, and Intentionality for Each Transgression Type

	Size of *More*/*Less* Asymmetry		Moral-Signaling Relevance (0-100)		Harmfulness (0-100)		Intentionality (0-100)	
Transgression Type	*b*	*SE*	Mean	*SD*	Mean	*SD*	Mean	*SD*
School shooting	0.25***	0.03	92.23	18.24	98.12	7.97	98.48	5.85
Sexual assault	0.23***	0.03	91.03	19.23	96.75	7.50	98.10	5.99
Vehicular manslaughter	0.18***	0.03	84.10	20.73	97.55	7.38	60.95	31.83
Knife attack	0.17***	0.03	88.01	19.81	97.42	7.29	96.72	9.00
Prescribing wrong medication	0.14***	0.03	63.92	29.04	91.19	12.62	43.57	31.15
Carjacking	0.12***	0.03	81.25	22.57	86.63	16.48	97.3	8.29
Mail theft	0.11**	0.03	65.56	27.34	69.66	23.37	94.88	10.45
Burglary	0.10***	0.03	77.05	24.50	79.58	21.61	97.09	7.24
Online harassment	0.09**	0.03	67.32	26.96	76.12	21.49	94.33	11.48
Bar fighting	0.04	0.03	59.08	28.60	77.49	20.31	85.14	17.78
Denting cars	0.04	0.03	63.05	28.43	64.38	24.55	75.19	27.21
Feeding children an allergen	0.02	0.04	62.69	29.18	88.81	16.8	45.09	32.64

Note: Results from Study 5. The second column shows the results of separate linear regressions testing for an effect of comparison frame (-0.5 = more, +0.5 = less) on whether participants assigned equal punishment to both cases for each pair of transgressions in the main study. The last three columns show mean ratings of moral-signaling relevance, harmfulness, and intentionality for each transgression type in the pretest. Transgression types are ordered in terms of the size of the *more*/*less* asymmetry (from largest to smallest).

Unless otherwise noted, all results reported below are based on linear regressions with the dependent variable predicted by order (−0.5 = less severe first, +0.5 = more severe first). For our primary analyses that test for the overall effects of order across all three transgression types, we include fixed effects for transgression type and cluster standard errors at the participant level.

#### Prison sentences

We began by testing for an order-based asymmetry in the absolute amount that participants adjusted prison sentences from one case to the next. For this analysis (and all other analyses based on raw punishment length responses), we excluded observations associated with unrealistically high values. As specified in our preregistrations, we flagged the highest 5% of punishment lengths assigned to the more severe case, across all participants and all three transgression types, and excluded observations associated with these flagged responses. In Study 2a, 19 out of 1,755 observations were flagged for exclusion (with punishments ranging from 110 years to 100,000,000 years); in Study 2b, 83 out of 1,752 observations were flagged for exclusion (with punishments ranging from 100 years to 9,999 years). We used this approach for two reasons. First, it limits noise associated with extreme responses, thus reducing the risk that our predicted asymmetry will emerge merely as an artifact of the response scale (given that open-ended response scales always leave more leeway to scale up than to scale down). Second, because this exclusion procedure places no restrictions on the range of possible responses from the start, it allows us to use participants’ raw judgments to capture other relevant indicators of adjustment besides absolute adjustment (e.g., proportional adjustment, and whether participants adjust at all).

**Fig. 2.**
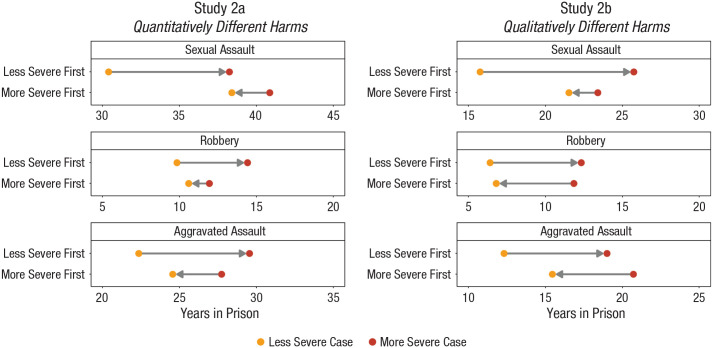
Adjustment of prison sentences between cases for each pair of transgressions in Studies 2a and 2b, as a function of order. Right-pointing arrows indicate upward adjustment from the less severe case to the more severe case, and left-pointing arrows indicate downward adjustment from the more severe case to the less severe case. The transgression pairs in Study 2a involved sexual assault of two versus three children, armed robbery of one versus several stores, and aggravated assault of one versus two people. The transgression pairs in Study 2b involved sexual assault of an adult versus a child, unarmed versus armed robbery, and aggravated assault causing cuts to the hands and arms versus stab wounds to the chest and abdomen. For most pairs, participants made larger upward than downward adjustments, consistent with our predicted asymmetry.

In both studies, we observed an overall order effect in line with our predicted asymmetry. In Study 2a, in which participants evaluated pairs of quantitatively different harms, those who started with the less severe case adjusted upward by an average of 6.52 years (*SD* = 9.24), whereas those who started with the more severe case adjusted downward by only 2.33 years (*SD* = 5.62), *b* = −4.21, clustered *SE* = 0.44, *t =* −9.52, *p* < .001. This asymmetry emerged within each of the three pairs of transgressions. In Study 2b, in which participants evaluated pairs of qualitatively different harms, those who started with the less severe case adjusted upward by an average of 7.46 years (*SD* = 9.92), whereas those who started with the more severe case adjusted downward by only 4.12 years (*SD* = 7.25), *b* = −3.33, clustered *SE* = 0.50, *t* = −6.65, *p* < .001. This asymmetry emerged for the pair of sexual-assault cases and the pair of aggravated-assault cases, but it did not emerge for the pair of robbery cases. Figure 2 displays the mean punishment length assigned to each case and the amount of adjustment between cases for each pair of transgressions.

In Study 2a, a similar order-based asymmetry emerged in ratio-based adjustment (which, as preregistered, involved no exclusions). The ratio between punishment lengths was smaller on average among participants who started with the more severe case (*M* = 1.17, *SD* = 0.39) than among those who started with the less severe case (*M* = 1.42, *SD* = 0.45), *b* = 0.25, clustered *SE* = 0.02, *t* = 11.38, *p* < .001. This pattern held for each of the three pairs of transgressions. In Study 2b, however, no asymmetry emerged in ratio-based adjustment overall, *b* = 0.38, clustered *SE* = 0.30, *t* = 1.26, *p* = .209, nor within any of the three transgression pairs. In the Supplemental Material, we report analyses of ratio-based adjustment in which we exclude extreme responses using the same exclusion rule that we preregistered for analyses of absolute adjustment. After exclusions, we found support for an asymmetry in ratio-based adjustment in Study 2b.

We also explored the frequency with which participants refrained from differentiating between cases altogether (i.e., assigned equal prison sentences to both cases). In both studies, participants differentiated between cases the majority of the time; however, in line with the predicted asymmetry, those who started with the more severe case refrained from differentiating more frequently than those who started with the less severe case. In Study 2a, participants who started with the less severe case assigned equal punishment to both cases only 23.4% of the time, whereas those who started with the more severe case did so 62.9% of the time, *b* = 1.72, odds ratio (*OR*) = 5.56, clustered *SE* = 0.13, *z* = 13.46, *p* < .001. In Study 2b, participants who started with the less severe case assigned equal punishment to both cases 15.0% of the time, whereas those who evaluated the more severe case first did so 40.0% of the time, *b* = 1.40, *OR* = 4.07, clustered *SE* = 0.13, *z* = 10.45, *p* < .001. This pattern held for each of the three transgression pairs in both studies.

#### Punishment ratings

Next, we tested for an order-based asymmetry on the scale-based punishment composite. Whereas the open-ended prison-sentence measure was unbounded in one direction, offering participants more leeway to adjust upward than downward, the scale-based measure was bounded on both ends (i.e., responses had to be between 1 and 11). In the vast majority of cases, participants started above the midpoint of the scale (85% of the time in Study 2a and 84% of the time in Study 2b), which means that they had more room to adjust downward than upward—biasing results against our predicted asymmetry. Nevertheless, in both studies, we found support for an order-based asymmetry on this measure. In Study 2a, those who started with the less severe case adjusted upward by an average of 0.38 (*SD* = 0.76) scale points, whereas those who started with the more severe case adjusted downward by only 0.18 points (*SD* = 0.61), *b* = −0.19, clustered *SE* = 0.03, *t* = −6.07, *p* < .001. This pattern held for each of the three transgression pairs. In Study 2b, participants who started with the less severe case adjusted upward by an average of 1.16 scale points (*SD* = 1.19), whereas those who started with the more severe case adjusted downward by only 0.80 points (*SD* = 1.21), *b* = −0.37, clustered *SE* = 0.06, *t* = −6.35, *p* < .001. This pattern emerged for the pair of sexual-assault cases and the pair of robbery cases, but not for the pair of aggravated-assault cases.

We observed a similar asymmetry in the frequency with which participants differentiated between cases at all. Participants in Study 2a who started with the less severe case assigned equal punishment to both cases 50.0% of the time, whereas those who started with the more severe case did so 66.2% of the time, *b* = 0.74, *OR* = 2.09, clustered *SE* = 0.12, *z* = 6.30, *p* < .001. This pattern held for each of the three transgression pairs. Participants in Study 2b who started with the less severe case assigned equal punishment to both cases 28.5% of the time, whereas those who started with the more severe case did so 42.4% of the time, *b* = 0.68, *OR* = 1.97, clustered *SE* = 0.13, *z* = 5.37, *p* < .001. This effect was significant only for the pair of sexual-assault cases. It was marginally significant for the pair of aggravated-assault cases and nonsignificant for the pair of robbery cases.

Across all transgression types in Studies 2a and 2b, the weakest support for our predicted asymmetry seemed to emerge for robberies. Although we designed and preregistered both studies to collapse across transgression types, we consider in the Supplemental Material several possible explanations for variation in the size of the asymmetry across transgression types. Study 5 directly investigates which features of a transgression best predict the size of the asymmetry.

In the Supplemental Material, we also report unplanned exploratory analyses that reveal a similar order-based asymmetry in the amount that participants adjusted between different pairs of transgressions that differed in severity (e.g., between the pair of sexual-assault cases and the pair of robberies), as a function of the random order in which they encountered each pair.

## Study 3

Study 3 tested for a directional asymmetry in simultaneous, rather than sequential, judgments. If an asymmetry emerges in sequential judgments because greater outrage at the more severe case spills over onto subsequent judgments of the less severe case, or because prior exposure to the more severe case changes which factors people consider when judging the less severe case, no asymmetry should emerge when both cases are presented simultaneously. In Study 3, participants encountered two side-by-side transgressions and judged which deserves more or less condemnation. Because the *more* frame demands scaling up, whereas the *less* frame demands scaling down, our account predicts that the asymmetry should persist.

### Method

#### Participants and design

We requested 600 participants from Prolific and received 601 complete submissions. Consistent with our preregistration, we excluded all submissions from participants who opened the survey more than once under the same participant ID or IP address (*n* = 8). The final sample consisted of 593 participants (48.6% men, 49.4% women, 2.0% other identity; mean age = 35.7 years). Participants were randomly assigned to one of two comparison frame conditions: *more* or *less*.

#### Procedure

To distinguish our account from alternative explanations specific to sequential judgment contexts, Study 3 manipulated the direction of comparison through framing. Specifically, we asked some participants to make upward (*more*) comparisons while asking others to make logically equivalent downward (*less*) comparisons. Whereas *more* comparisons (e.g., “B is more wrong than A”) allow for the possibility that both cases are bad, *less* comparisons (e.g., “A is less wrong than B”) may imply that one case is not very bad (Inbar & Evers, 2022). We thus expected that participants in the *less* condition would be more likely to condemn both cases equally than those in the *more* condition, given that only *less* comparisons could be construed as downplaying.

Participants considered five pairs of transgressions, each of which included a less severe case and a more severe case: a pair of aggravated-assault cases (attacking one vs. two elderly people), a pair of kidnapping cases (kidnapping two vs. four children), a pair of manslaughter cases (killing one vs. three people), a pair of robbery cases (robbery of one vs. several stores), and a pair of sexual-assault cases (assaulting an adult victim vs. a child victim). Both cases in a given pair were presented side by side on the same page. We randomized which case appeared on which side of the page.

For each pair of transgressions, participants were asked to make two comparative judgments, one about punishment and the other about moral wrongness. Those in the *more* condition were always asked, “Which of these two cases should be PUNISHED MORE SEVERELY?” and “Which of these two cases is MORE MORALLY WRONG?” Meanwhile, those in the *less* condition were always asked, “Which of these two cases should be PUNISHED LESS SEVERELY?” and “Which of these two cases is LESS MORALLY WRONG?”

Each question offered three response options that reiterated the relevant comparison frame. Participants could indicate that the more severe case deserved more condemnation (e.g., “the perpetrator in CASE 1 (Person A) should receive MORE punishment” in the *more* condition or “the perpetrator in CASE 2 (Person B) should receive LESS punishment” in the *less* condition), that the less severe case deserved more condemnation (e.g., “The perpetrator in CASE 2 (Person B) should receive MORE punishment” in the *more* condition or “The perpetrator in CASE 1 (Person A) should receive LESS punishment” in the *less* condition), or that both cases deserved equal condemnation (e.g., “Both perpetrators should be punished EQUALLY” in both conditions). Condemning both cases equally can be interpreted as choosing not to differentiate between those cases according to severity.

### Results

Our primary dependent variables captured whether participants chose not to differentiate between cases according to severity. We created an equal-punishment variable with a value of 1 if participants said both cases in a given pair should be punished equally and 0 otherwise, and an equal-wrongness variable with a value of 1 if they said both cases were equally wrong and 0 otherwise. For each dependent measure, we conducted a linear regression with comparison frame (−0.5 = more, +0.5 = less) as a predictor, including fixed effects for each pair of transgressions and clustering standard errors by participant. As specified in our preregistration, we used linear regression because it yields more directly interpretable coefficients (Gomila, 2021). However, results are essentially identical using logistic regression.

#### Punishment

**Fig. 3.**
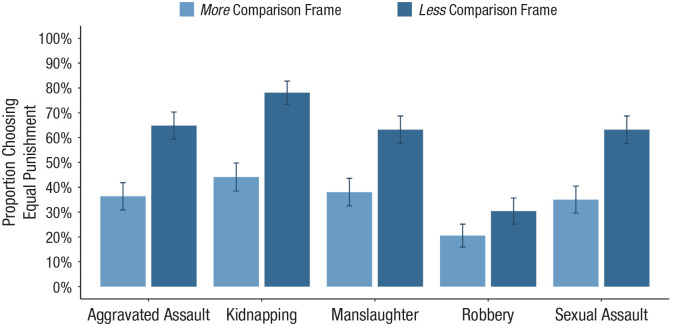
Proportion of participants in Study 3 who endorsed punishing a given pair of transgressions equally as a function of whether they were asked which case should be punished more severely or less severely. Comparisons involved a pair of aggravated-assault cases (one vs. two victims), a pair of kidnapping cases (two vs. four victims), a pair of manslaughter cases (one vs. three victims), a pair of robbery cases (one vs. several stores), and a pair of sexual-assault cases (adult victim vs. child victim). Error bars represent 95% confidence intervals.

Relative to participants in the *more* condition, those in the *less* condition assigned equal punishment to both cases significantly more often, *b* = 0.25, *SE* = 0.03, *t* = 9.23, *p* < .001. Overall, those who were asked which case should be punished more severely assigned equal punishment to both cases 34.8% of the time, choosing the more severe case—the logically appropriate option—63.8% of the time. By contrast, those who were asked which case should be punished less severely assigned equal punishment to both cases nearly twice as often (59.9%) and chose the less severe case—the logically appropriate option—less frequently (38.6%). Participants rarely indicated that the less severe case deserved more punishment, regardless of whether they were in the *more* (1.4%) or *less* (1.5%) condition, suggesting that our results are not driven by misunderstanding of either comparison frame. Figure 3 displays the proportion of participants who assigned equal punishment to both cases for each pair of transgressions.

#### Moral wrongness

We observed a similar pattern in participants’ moral wrongness judgments. Participants in the *less* condition indicated that both cases were equally wrong significantly more often than those in the *more* condition, *b* = 0.08, *SE* = 0.02, *t* = 3.76, *p* < .001. Those who were asked which case was more wrong indicated that both cases were equally wrong 77.1% of the time and chose the more severe case 22.6% of the time. Those who were asked which case was less wrong indicated that both cases were equally wrong 85.3% of the time and chose the less severe case only 13.5% of the time. Participants rarely indicated that the less severe case was more wrong, although those in the *less* condition did so somewhat more frequently (1.2%) than those in the *more* condition (0.3%), *b* = 0.01, *SE* = 0.004, *t* = 2.06, *p* = .040.

In sum, Study 3 demonstrates that a directional asymmetry also emerges in simultaneous judgments. Just as participants in Studies 1, 2a, and 2b differentiated between transgressions more when starting with the less severe case than when starting with the more severe case, participants in Study 3 were more likely to differentiate when asked which case deserved more condemnation than when asked which case deserved less condemnation.

## Study 4

Study 4 examined whether the asymmetry is smaller when judging how much punishment a pair of transgressions would receive than when judging how much punishment those transgressions should receive. Although both types of judgments involve assigning punishment to a pair of transgressions, *would* judgments are predictions about what will happen and thus do not implicate moral character as strongly as *should* judgments. If people are simply less likely to recognize differences in severity in downward comparison contexts, then the asymmetry should be just as large when judging what would happen as when judging what should happen. However, if the asymmetry is rooted in a desire to avoid expressing insufficient condemnation, then it should be larger for *should* (vs. *would*) judgments.

### Method

#### Participants and design

We requested 1,600 participants from Prolific and received 1,613 complete submissions. Consistent with our preregistration, we excluded all submissions from participants who opened the survey more than once under the same participant ID or IP address (*n* = 40). The final sample consisted of 1,573 participants (49.6% men, 48.8% women, 1.7% other identity; mean age = 40.0 years). Participants were randomly assigned to one of four conditions in a 2 (judgment type: *should* vs. *would*) × 2 (comparison frame: *more* vs. *less*) between-subjects design.

#### Procedure

All participants evaluated a pair of manslaughter cases in which drivers under the influence of alcohol struck and killed different numbers of pedestrians. The less severe case involved one victim, and the more severe case involved three victims. As in Study 3, both cases were shown side by side, and we randomized which case appeared on which side of the page.

Participants were asked to compare the two cases in one of two ways. Those in the *should* condition were prompted to “consider how much punishment each case should receive based on YOUR PERSONAL FEELINGS,” whereas those in the *would* condition were prompted to “consider how much punishment each case would receive in a COURT OF LAW.”

We used the same comparison-frame manipulation as in Study 3. In the *more* condition, participants were asked which case should (or would) receive more punishment, whereas in the *less* condition they were asked which case should (or would) receive less punishment. Those in the *should* condition could indicate that the more severe case should receive more punishment (by choosing the more severe case in the *more* condition or the less severe case in the *less* condition), that the less severe case should receive more punishment (by choosing the less severe case in the *more* condition or the more severe case in the *less* condition), or that both cases should receive equal punishment. In the *would* condition, the response options were identical except that the word “should” was replaced by “would.”

At the end of the survey, participants answered an additional question to assess whether they had internalized the manipulation. Specifically, they were asked to recall whether they had been instructed to consider “how much punishment each case should receive based on YOUR PERSONAL FEELINGS” or “how much punishment each case would receive in a COURT OF LAW.” Nearly all participants answered correctly, and there was no detectable difference in the proportion who answered correctly in the *should* condition (95.2%) versus the *would* condition (96.0%), χ^2^(1, *N* = 1,573) = 0.66, *p* = .416.

#### Posttest

To confirm that *should* judgments are seen as more indicative of one’s moral character than *would* judgments, we conducted a posttest in which participants (*N* = 393) were asked to imagine making a judgment about how much punishment a pair of criminal offenses either should receive (“based on YOUR PERSONAL FEELINGS”) or would receive (“in a COURT OF LAW”). Participants rated the extent to which the target judgment would implicate their moral character: “To what extent would your answer say something about your moral character?” (0 = *not at all*, 10 = *very much*).

### Results

#### Posttest

In line with our assumption that *should* judgments more strongly implicate moral character than *would* judgments, posttest participants indicated that a judgment of how much punishment a pair of criminal offenses should receive would say more about their moral character (*M* = 8.03, *SD* = 1.84) than a judgment of how much punishment a pair of criminal offenses would receive (*M* = 7.01, *SD* = 2.63), *t*(391) = 4.44, *p* < .001, *d* = 0.45.

#### Punishment

Consistent with our preregistration, we recoded participants’ choices to indicate whether they assigned equal punishment to both cases, which would mean that they did not differentiate between the case involving one victim and the case involving three victims. The equal-punishment variable was coded as 1 if a participant assigned equal punishment to both cases and 0 otherwise.

We conducted a linear regression with equal punishment predicted by judgment type (−0.5 = would, +0.5 = should), comparison frame (−0.5 = more, +0.5 = less), and their two-way interaction. Although we did not predict an overall difference between *should* and *would* judgments, we found that participants were more likely to assign equal punishment when they were asked to judge how much punishment each case should receive than when they were asked to judge how much punishment each case would receive, *b* = 0.22, *SE* = 0.02, *t*(1569) = 10.41, *p* < .001. In other words, participants differentiated less on the basis of severity when expressing personal condemnation than when reporting what they would expect to happen in a court of law.

Replicating the asymmetry we observed in Study 3, participants who were prompted to make a *less* comparison were more likely to assign equal punishment to both cases than those who were prompted to make a *more* comparison, *b* = 0.11, *SE* = 0.02, *t*(1569) = 5.41, *p* < .001. However, as we predicted, the size of this asymmetry depended on whether participants were asked to make a *should* or *would* judgment, *b* = 0.15, *SE* = 0.04, *t*(1569) = 3.47, *p* < .001.

**Fig. 4.**
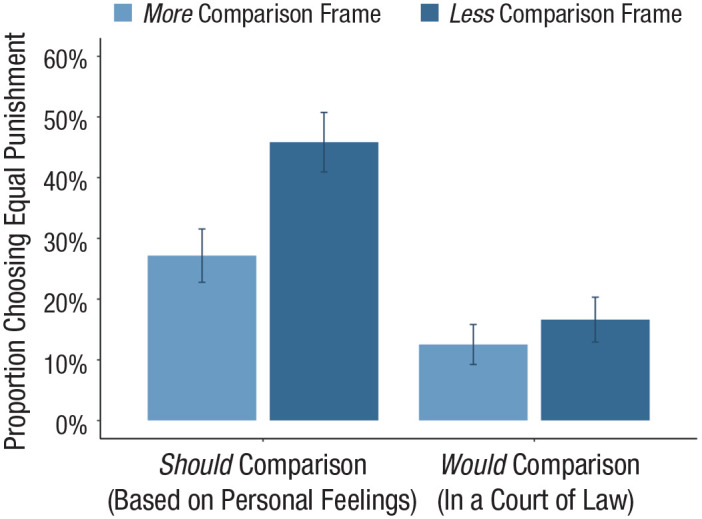
Proportion of participants in Study 4 who assigned equal punishment to a pair of manslaughter cases (involving one vs. three victims) as a function of whether they were asked to consider how much punishment each case *should* or *would* receive and whether they were asked to make a *more* or *less* comparison. Participants in the *should* condition refrained from differentiating between cases more often when making a *less* (vs. *more*) comparison, but this asymmetry disappeared in the *would* condition. Error bars represent 95% confidence intervals.

To follow up, we examined the simple effect of the *less* (vs. *more*) comparison frame in the *should* and *would* conditions separately. In the *should* condition, we observed a large directional asymmetry: Participants who were asked which case should receive less punishment selected equal punishment significantly more often (45.8%) than those who were asked which case should receive more punishment (27.2%), *b* = 0.19, *SE* = 0.03, *t*(1569) = 6.29, *p* < .001. In the *would* condition, this asymmetry disappeared: Participants who were asked which case would receive less punishment were no more likely to select equal punishment (16.6%) than those who were asked which case would receive more punishment (12.5%), *b* = 0.04, *SE* = 0.03, *t*(1569) = 1.37, *p* = .171. Results are displayed in Figure 4.

Altogether, the results of Study 4 supported our prediction that a larger *more*/*less* asymmetry would emerge when participants were asked to judge how much punishment a pair of transgressions should receive than when they were asked to judge how much punishment those transgressions would receive. Whereas both *should* and *would* judgments involve assigning punishment to the same two transgressions, *should* judgments implicate moral character to a greater extent.

These findings are consistent with our reluctance-to-downplay account but inconsistent with several alternative accounts that are not specific to moral judgment—for example, that people are less likely to perceive differences in severity in downward (vs. upward) comparison contexts (e.g., Fechner, 1948; Weber, 1996) or find *less* (vs. *more*) comparisons more difficult (e.g., Alter & Oppenheimer, 2009; Novemsky et al., 2007). Yet one possibility we cannot rule out is that *should* judgments yield a larger asymmetry simply because they seem more subjective than *would* judgments. Although the extent to which a judgment is subjective and the extent to which it implicates moral character are conceptually distinct, we suspect that they would be difficult to disentangle empirically. Given the inherent difficulty of controlling for all possible differences between different types of judgments, Study 5 instead asks how the size of the asymmetry varies for different types of transgressions.

## Study 5

Our reluctance-to-downplay account predicts that people will resist scaling down condemnation more when doing so calls their moral character into question to a greater extent. Given that some transgressions are seen as more important to condemn than others, in Study 5 we investigated whether variation in *moral-signaling relevance* (i.e., how much a failure to condemn signals bad moral character) predicts variation in the size of the asymmetry across different types of transgressions. One group of participants rated the features of several transgression types (e.g., theft, assault, murder), including their moral-signaling relevance, and another group of participants made *more* or *less* comparisons for each transgression type.

### Method

#### Participants and design

We requested 800 participants from Prolific and received 799 complete responses. Consistent with our preregistration, we excluded all submissions from participants who opened the survey more than once under the same participant ID or IP address (*n* = 12). The final sample consisted of 787 participants (49.4% men, 49.3% women, 1.3% other identity; mean age = 40.4 years). Participants were assigned to one of two comparison-frame conditions: *more* or *less*.

#### Procedure

Participants in the main study evaluated 12 pairs of transgressions drawn from the following categories: school shooting, sexual assault, knife attack, vehicular manslaughter, carjacking, burglary, online harassment, doctors prescribing patients the wrong medication, teachers feeding children an allergen, bar fighting, and mail theft. We expected these transgression types to vary in moral-signaling relevance (i.e., the extent to which a failure to condemn signals bad moral character), as well as in their perceived harmfulness and intentionality.

For each type of transgression, we varied the number of victims to create a less severe case (involving one victim) and a more severe case (involving three victims). For each pair of transgressions, participants were asked to make either an upward (*more*) comparison or a downward (*less*) comparison. As in Study 3, they chose from three response options: assigning more punishment to the more severe case (by choosing the more severe case in the *more* condition or the less severe case in the *less* condition), assigning more punishment to the less severe case (by choosing the less severe case in the *more* condition or the more severe case in the *less* condition), or assigning equal punishment to both cases. Both cases were presented side by side on the same page, and we randomized which case appeared on which side of the page. The 12 transgression pairs were presented in random order, one pair per page.

#### Pretest

Before the main study, we conducted a pretest to measure perceptions of each transgression type’s moral-signaling relevance, harmfulness, and intentionality, which could then be used to predict the size of the asymmetry in the main study. Pretest participants (*N* = 385) were presented with the same 12 transgression types used in the main study (in random order), but without reference to a specific number of victims. For example, online harassment was described as “repeatedly harassing one or more people on the internet with threatening messages.” This ensured that participants’ judgments would be based only on the general features of each transgression type and not on the details of any particular case.

For each transgression type, participants were asked, “Consider someone who is not concerned about this offense. To what extent are they a bad person?” (0 = *a not at all bad person*, 100 = *an extremely bad person*); “To what extent is this offense harmful?” (0 = *not at all harmful*, 100 = *extremely harmful*); and “To what extent is this offense intentional?” (0 = *not at all intentional*, 100 = *extremely intentional*). The three measures for each transgression type were presented in a random order, and one measure was presented on each page.

### Results

Our central question was whether variation in moral-signaling relevance, harmfulness, and intentionality across the 12 transgression types (as judged by pretest participants) would predict variation in the size of the *more*/*less* asymmetry in the main study. Of course, given that transgressions seen as more harmful or intentional may also seem more important to condemn, the size of the asymmetry may vary with any or all of these features (as well as others). However, we sought to explore whether any feature was particularly predictive.

#### Pretest

In the pretest, ratings of moral-signaling relevance were positively correlated with ratings of harmfulness (*r* = .509) and intentionality (*r* = .341). Ratings of harmfulness and intentionality were positively correlated (*r* = .088), though to a lesser extent. We used these pretest ratings to calculate the average moral-signaling relevance, harmfulness, and intentionality rating for each transgression type, rounded to two decimal places (Table 2).

#### Main study

Our primary dependent variable was whether participants assigned equal punishment to both cases in a pair—in other words, refrained from differentiating between cases involving one versus three victims. Overall, in line with the asymmetry observed in Study 3, participants assigned equal punishment to both cases more frequently when asked which case deserved less punishment (39.5%) than when asked which case deserved more punishment (27.2%), *b* = 0.12, *SE* = 0.02, *t*(785.00) = 5.91, *p* < .001. Unsurprisingly, participants differentiated between cases at different rates for different types of transgressions; for instance, they differentiated between two cases of burglary more frequently than they differentiated between two cases of vehicular manslaughter. However, we were primarily interested in how the size of the asymmetry varied across transgression types.

**Fig. 5.**
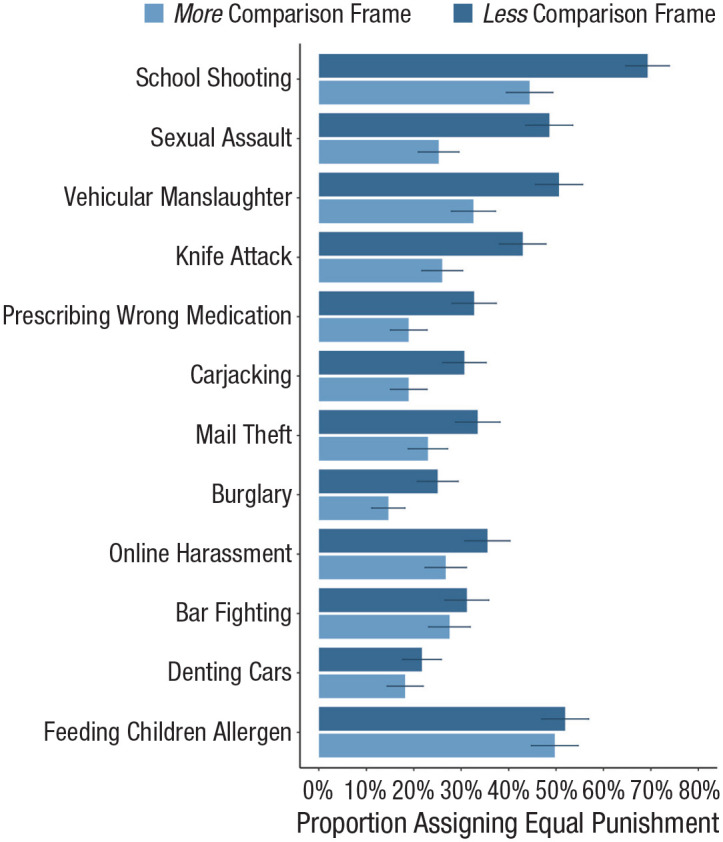
Proportion of participants who assigned equal punishment to both cases as a function of whether they were asked to make a *more* or *less* comparison, for each transgression type in Study 5. Error bars represent 95% confidence intervals.

As shown in Figure 5, the asymmetry was larger for some types of transgressions (e.g., school shootings, sexual assaults) than for others (e.g., denting cars, teachers feeding children an allergen). To probe which feature best predicted the size of the asymmetry, we began by exploring the correlation between the size of the asymmetry and each potential moderator across transgression types. To do so, we assigned ranks to each transgression type according to the size of the asymmetry for the pair of transgressions of that type in the main study and the average ratings of moral-signaling relevance, harmfulness, and intentionality for that transgression type in the pretest (Table 2), with higher ranks indicating larger values.

**Fig. 6.**
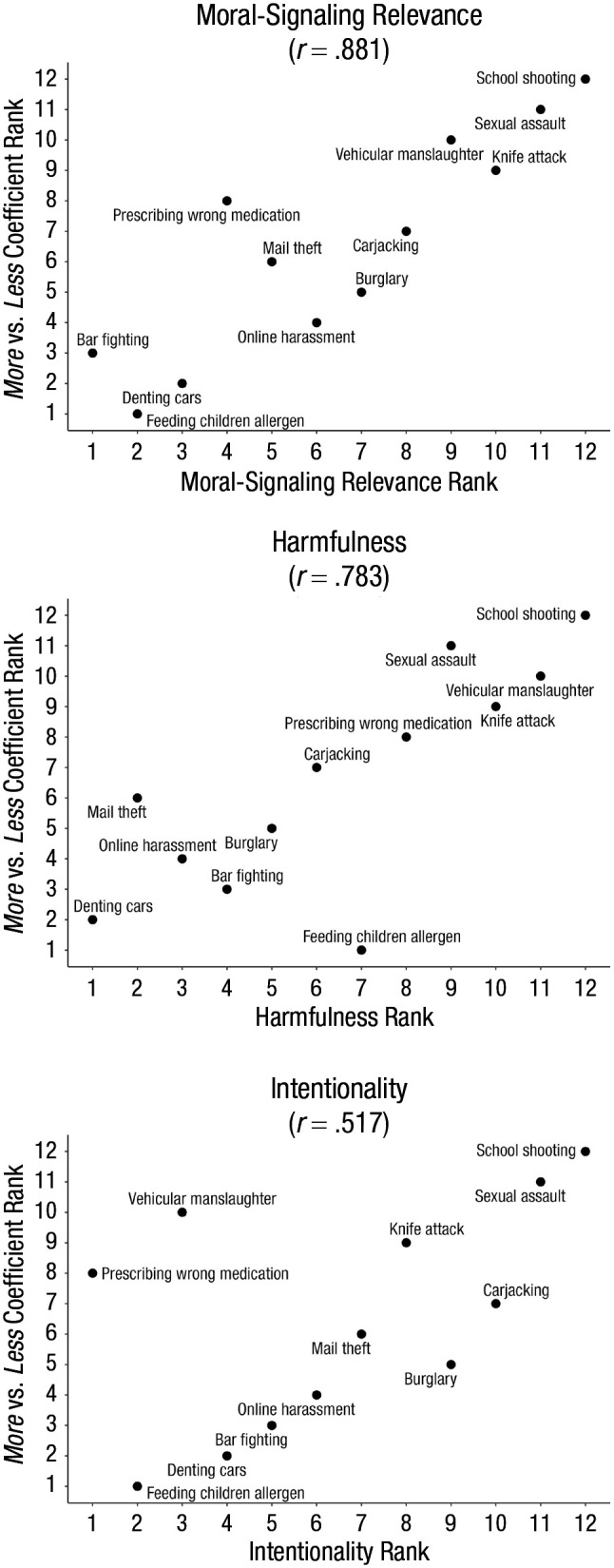
Association between each transgression type’s ranking on each potential moderator (in the pretest) and the size of the *more/less* asymmetry for transgressions of that type (in the main study) in Study 5. Higher ranks indicate larger values.

Across the 12 transgression types, the size of the *more/less* asymmetry was most strongly associated with a transgression type’s perceived moral-signaling relevance, *r* = .881, *p* < .001, followed by its perceived harmfulness, *r* = .783, *p* = .003, followed by its perceived intentionality, *r* = .517, *p* = .085 (Fig. 6). For example, feeding children an allergen—which yielded the smallest asymmetry—was rated lower on moral-signaling relevance than nearly all other types of transgressions, despite being perceived as more harmful than most other types of transgressions. Meanwhile, vehicular manslaughter—which yielded the third-largest asymmetry—was rated higher on moral-signaling relevance than most other transgressions, despite being perceived as one of the least intentional types of transgressions. Similar patterns emerged in the correlations between the raw regression coefficients and the average transgression-type-level ratings across the 12 transgression types, which are reported in the Supplemental Material. There, we also report tests of the differences between these correlations, which we treat as exploratory given that they are based on only 12 observations each (one per transgression type). As preregistered, the primary way we planned to test for differences between potential moderators was by comparing participant-level regression models.

For our primary analyses, we first tested for an interaction between the size of the *more*/*less* asymmetry and each potential moderator at the participant level. To do so, we conducted three linear mixed-effects regressions, each of which included terms for the main effects of comparison frame, moral-signaling relevance, harmfulness, and intentionality, and a single interaction term. All models included participant-level random intercepts to account for the fact that each participant provided 12 observations (one for each transgression type). The interaction term in each model tested for a two-way interaction between comparison frame and one of the three potential moderators (moral-signaling relevance, harmfulness, or intentionality). Although we specified in our preregistration that we would use logistic regression, we instead report linear regression models because the logistic regression models failed to converge. Results of each model and indices of model fit are displayed in Table 3. The interaction term was significant in all three models, indicating that individually, all three features that we measured in the pretest were related to the size of the asymmetry. In other words, the asymmetry was larger for types of transgressions that were seen as more important to condemn, those perceived as more harmful, and those perceived as more intentional.

**Table 3.** Results of the Mixed-Effects Regression Models Used to Compare Potential Moderators of the *More*/*Less* Asymmetry in Study 5

	Moral-signaling relevance as moderator	Harmfulness as moderator	Intentionality as moderator
Predictor	(1)	(2)	(3)
Intercept	0.3333*** (0.0104)	0.3333*** (0.0104)	0.3333*** (0.0104)
Comparison frame (−0.5 = more, +0.5 = less)	0.1233*** (0.0209)	0.1233*** (0.0209)	0.1233*** (0.0209)
Moral-signaling relevance (mean-centered)	−0.0012 (0.0008)	−0.0012 (0.0008)	−0.0012 (0.0008)
Perceived harmfulness (mean-centered)	0.0072*** (0.0008)	0.0072*** (0.0008)	0.0072*** (0.0008)
Perceived intentionality (mean-centered)	−0.0001 (0.0003)	−0.0001 (0.0003)	−0.0001 (0.0003)
Comparison Frame × Moral-Signaling Relevance	0.0054*** (0.0006)	**—**	**—**
Comparison Frame × Harmfulness	**—**	0.0047*** (0.0007)	**—**
Comparison Frame × Intentionality	**—**	**—**	0.0012** (0.0004)
Observations	9444	9444	9444
Participants	787	787	787
Akaike Information Criterion (AIC)	**9,778.210**	9,798.647	9,837.386
Bayesian Information Criterion (BIC)	**9,835.435**	9,855.872	9,894.611
Marginal/conditional *R*^2^	0.045/0.378	0.043/0.376	0.041/0.373

Note: This table shows results of the three linear mixed-effects regressions we used to compare potential moderators of the *more*/*less* asymmetry. Each model regressed the decision to assign equal punishment to the more severe and less severe case within a pair of transgressions (1 = yes, 0 = no) on (1) comparison frame (−0.5 = more, +0.5 = less), (2–4) the mean pretest ratings of the transgression type’s moral-signaling relevance, harmfulness, and intentionality (mean-centered), and (5) the interaction between comparison frame and one of the three potential moderators. All models include participant-level random intercepts. The only difference between models was which of the three potential moderators was included in the interaction term. Boldface indicates the lowest Akaike information criterion (AIC) and Bayesian information criterion (BIC) values, indicating the best fit. **p < .01. ***p < .001.

Next, to examine which feature best predicted the size of the asymmetry, we compared model fit indices across the three models. Because the only difference between models was which potential moderator we included in the interaction term, the model that best fits the data should reveal which feature most strongly predicts the size of the asymmetry. Consistent with our preregistration, we assessed model fit using the Akaike information criterion (AIC; Akaike, 1974) and the Bayesian information criterion (BIC; Schwarz, 1978), with lower values indicating better fit. On both indices, the model that included a term for the interaction between comparison frame and moral-signaling relevance best fit the data (Table 3).

Taken together, the results suggest that people scale up and down asymmetrically to a greater extent when comparing transgressions for which expressing condemnation is seen as a stronger signal of moral character. Although the asymmetry was also larger for more harmful and intentional types of transgressions, moral-signaling relevance was a more reliable predictor than either of these features alone, as predicted by our reluctance-to-downplay account.

It is important to note that this does not mean that perceptions of harmfulness and intentionality are unrelated to the size of the asymmetry. Rather, these features may simply be components of what makes a particular wrongdoing seem especially important to condemn. In Supplemental Study 2 in the Supplemental Material, we experimentally tested whether the asymmetry is larger for more harmful pairs of transgressions, using more controlled stimuli in which the perpetrators’ actions are held constant across pairs (e.g., nurses failing to give patients their medication). Indeed, an order-based asymmetry emerged for pairs of transgressions that caused serious harm (e.g., death), but no asymmetry emerged for pairs of similar transgressions that caused only minor harm (e.g., headaches). These results support the idea that one of the features that can make a transgression seem especially important to condemn is its perceived harmfulness, as suggested by Study 5.

## Study 6

We have proposed that people resist scaling down condemnation because indicating that one transgression deserves less condemnation than another can be construed as downplaying, a signal of bad moral character. Study 6 investigated whether observers’ judgments support this assertion. We expected that observers would penalize someone who says that the less severe of two transgressions deserves less punishment (vs. condemning both equally), yet reward someone who says that the more severe transgression deserves more punishment. Although such a pattern would not conclusively show that the asymmetry arises solely due to concerns about social reputation, it would suggest that the asymmetry is at least reinforced by social incentives.

### Method

#### Participants and design

We requested 800 participants from Prolific and received 799 complete submissions. Consistent with our preregistration, we excluded all submissions from participants who opened the survey more than once under the same participant ID or IP address (*n* = 14). The final sample consisted of 785 participants (48.7% men, 49.2% women, 2.2% other identity; mean age = 38.8 years). Participants were randomly assigned to one of four conditions in a 2 (comparison frame: *more* vs. *less*) × 2 (differentiation: *did differentiate* vs. *did not differentiate*) between-subjects design.

#### Procedure

Participants considered a scenario in which a target (whose name was randomized to be either “John,” “Lisa,” “Nora,” or “Owen”) was discussing two recent cases of celebrity sexual misconduct with a group of acquaintances. One case was transparently more severe than the other: The less severe case described a celebrity who was accused of sexually assaulting four women between the ages of 29 and 40, and the more severe case described a celebrity who was accused of sexually assaulting 30 girls and women between the ages of 14 and 37. The two cases were presented side by side. We randomized which case appeared on which side of the page, with the perpetrator on the left labeled “Celebrity A” and the perpetrator on the right labeled “Celebrity B.”

In the scenario, someone in the group asked the target to make either an upward comparison (*more* condition) or a downward comparison (*less* condition) between the two cases. In the *more* condition, the target was asked, “Do you think Celebrity B deserves more punishment than Celebrity A?” Meanwhile, in the *less* condition, the target was asked, “Do you think Celebrity A deserves less punishment than Celebrity B?”^2^

The target then responded by either differentiating or not differentiating between the two cases. In the *more* condition, the differentiating response was, “Yes, I think Celebrity B deserves much more punishment than Celebrity A,” whereas the nondifferentiating response was, “No, I don’t think Celebrity B deserves any more punishment than Celebrity A.” In the *less* condition, the differentiating response was, “Yes, I think Celebrity A deserves much less punishment than Celebrity B,” and the nondifferentiating response was, “No, I don’t think Celebrity A deserves any less punishment than Celebrity B.”

Participants evaluated the moral character of the target (in this example, “John”) on three dimensions ranging from the most broad to the most specific: (a) perceived morality: “To what extent is John a moral person?”; (b) perceived concern about women’s rights: “To what extent does John care about women’s rights?”; and (c) perceived concern about sexual misconduct: “To what extent is John concerned about sexual misconduct?” Responses were elicited on a 7-point scale (1 = *not at all*, 7 = *extremely*). The three items were presented in a fixed order, one per page.

Finally, participants completed an attention-check measure that asked them to recall which celebrity was involved in which case. Consistent with our preregistration, we conducted our primary analysis on the full sample, including participants who failed this attention check (*n* = 11); however, none of our key results change if we exclude those who failed the attention check.

### Results

We predicted that a target who was prompted to make an upward comparison would be evaluated more favorably if they differentiated between cases (i.e., if they expressed more condemnation of the more severe case), whereas a target who was prompted to make a downward comparison would be evaluated more favorably if they did not differentiate (i.e., if they expressed no less condemnation of the less severe case). We regressed each dependent variable on comparison frame (−0.5 = more, +0.5 = less), differentiation (−0.5 = did not differentiate, +0.5 = did differentiate), and the two-way interaction between these variables.

Overall, the target was evaluated more favorably when prompted to make a *less* (vs. a *more*) comparison. This effect emerged for judgments of the target’s morality, *b* = 0.25, *SE* = 0.10, *t*(781) = 2.54, *p* = .011, the extent to which they cared about women’s rights, *b* = 0.25, *SE* = 0.10, *t*(781) = 2.43, *p* = .016, and the extent to which they were concerned about sexual misconduct, *b* = 0.39, *SE* = 0.10, *t*(781) = 3.77, *p* < .001. The target was also evaluated more favorably if they differentiated (vs. did not differentiate) between transgressions. This effect emerged for judgments of the target’s morality, *b* = 0.59, *SE* = 0.10, *t*(781) = 6.09, *p* < .001, the extent to which they cared about women’s rights, *b* = 0.30, *SE* = 0.10, *t*(781) = 3.00, *p* = .003, and the extent to which they were concerned about sexual misconduct, *b* = 0.51, *SE* = 0.10, *t*(781) = 5.01, *p* < .001.

**Fig. 7.**
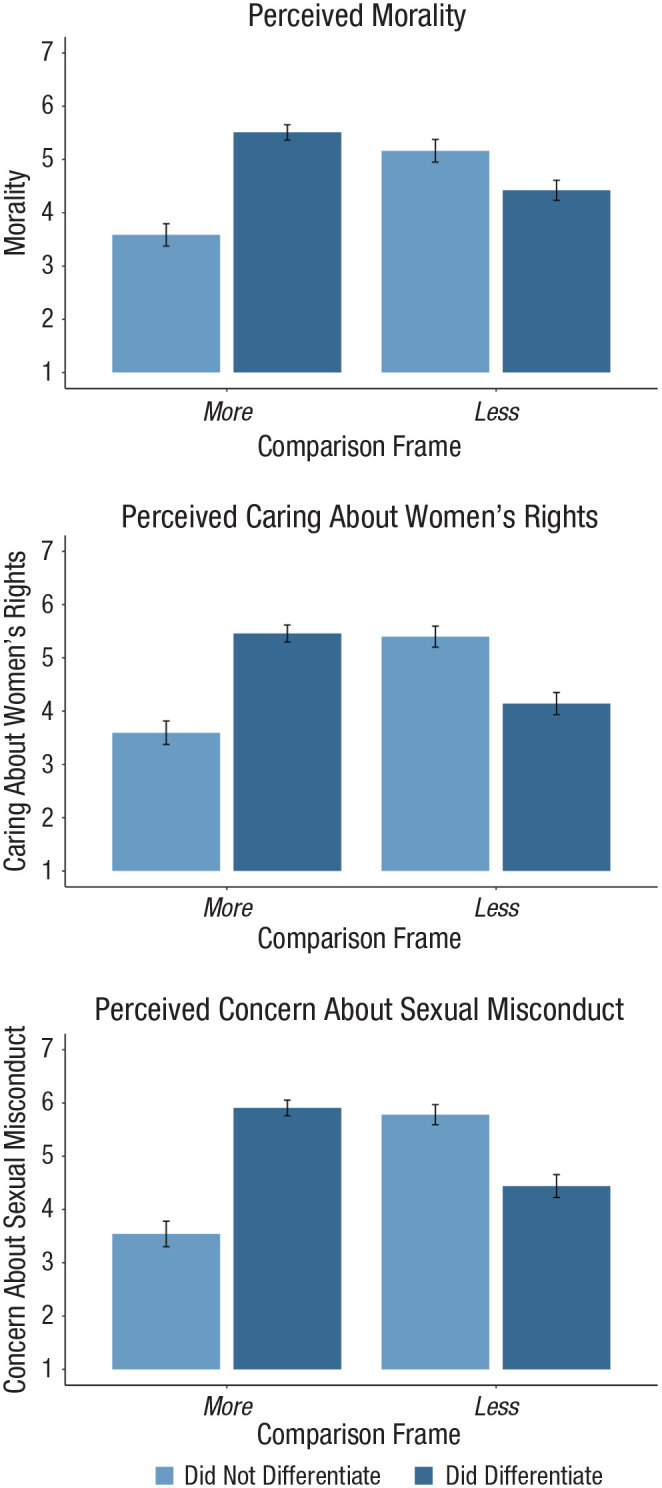
Results from Study 6. This figure shows judgments on a scale from 1 to 7 of a target’s morality (top), the extent to which the target cares about women’s rights (middle), and the extent to which the target is concerned about sexual misconduct (bottom), as a function of whether the target was prompted to make a *more* or *less* comparison between two sexual-misconduct cases and whether they did or did not differentiate between cases. Error bars represent 95% confidence intervals.

Most relevant to our central hypotheses, these main effects were qualified by a significant interaction in all three models (Fig. 7). In other words, differentiating (vs. not differentiating) between transgressions had a different effect on moral-character judgments in the *less* (vs. *more*) comparison frame. A similar interaction pattern emerged for judgments of the target’s morality, *b* = −2.67, *SE* = 0.19, *t*(781) = −13.74, *p* < .001, the extent to which they cared about women’s rights, *b* = −3.12, *SE* = 0.20, *t*(781) = −15.44, *p* < .001, and their concern about sexual misconduct, *b* = −3.70, *SE* = 0.21, *t*(781) = −18.09, *p* < .001. We followed up by examining the simple effect of differentiating (vs. not differentiating) within each comparison frame condition.

When prompted to make a *more* comparison, the target was rated as more moral if they differentiated between the two misconduct cases (*M* = 5.51, *SD* = 1.04) than if they did not differentiate (*M* = 3.58, *SD* = 1.49), *b* = 1.92, *SE* = 0.14, *t*(781) = 14.01, *p* < .001. Likewise, if they differentiated between cases, they were perceived as caring more about women’s rights (*M_did differentiate_* = 5.46, *SD* = 1.14; *M_did not differentiate_* = 3.59, *SD* = 1.58), *b* = 1.86, *SE* = 0.14, *t*(781) = 13.03, *p* < .001, and as being more concerned about sexual misconduct (*M_did differentiate_* = 5.91, *SD* = 1.05; *M_did not differentiate_* = 3.54, *SD* = 1.70), *b* = 2.37, *SE* = 0.15, *t*(781) = 16.33, *p* < .001. By contrast, when prompted to make a *less* comparison, the target was rated as less moral if they differentiated between cases (*M* = 4.42, *SD* = 1.35) than if they did not differentiate (*M* = 5.16, *SD* = 1.51), *b* = −0.74, *SE* = 0.14, *t*(781) = −5.41, *p* < .001. If they differentiated between cases, they were also perceived as caring less about women’s rights (*M_did differentiate_* = 4.14, *SD* = 1.48; *M_did not differentiate_* = 5.40, *SD* = 1.41), *b* = −1.26, *SE* = 0.14, *t*(781) = −8.80, *p* < .001, and as being less concerned about sexual misconduct (*M_did differentiate_* = 4.44, *SD* = 1.55; *M_did not differentiate_* = 5.78, *SD* = 1.35), *b* = −1.34, *SE* = 0.15, *t*(781) = −9.26, *p* < .001.

In sum, the results of this study demonstrate that scaling down condemnation can have reputational costs, even in a context in which scaling up offers reputational benefits. This pattern of moral-character evaluations aligns with the asymmetry we observed in individuals’ condemnation judgments in Studies 1 through 5. Note that these results do not necessarily imply that the asymmetry arises solely because of concerns about social reputation. Nevertheless, this study suggests that the asymmetry is at least reinforced by social incentives.

## General Discussion

This research demonstrates that the extent to which people differentiate between bad acts hinges on a seemingly irrelevant factor: the direction of comparison. Participants readily differentiated between transgressions when scaling up condemnation from a less severe case to a more severe one, but they differentiated much less—and often not at all—when scaling down. This directional asymmetry emerged for both real-world misconduct cases and controlled pairs of transgressions. In line with our reluctance-to-downplay account, it was especially pronounced for judgments that more strongly implicate moral character and for transgressions that seem more important to condemn. The asymmetry also manifested in observers’ moral-character judgments, suggesting that it is reinforced by social incentives.

We documented this directional asymmetry in both sequential and simultaneous judgment contexts, yet each method has limitations. In the sequential judgment paradigm used in Studies 1 through 2b, people may adjust asymmetrically from one case to the next because prior exposure to a more serious transgression changes how they feel or think about subsequent transgressions. Baron and Spranca (1997), observing a similar order effect, posited that the outrage evoked by one action may spill over onto judgments of subsequent actions in sequential evaluation contexts. To help distinguish our reluctance-to-downplay account from such alternatives, we moved to a simultaneous judgment context.

In Studies 3 through 5, we asked participants to judge which of two side-by-side transgressions deserves more or less punishment. Presenting both cases simultaneously holds potential outrage spillover constant across conditions, and it avoids other scale-related issues discussed in Studies 2a and 2b. However, it also raises new alternative explanations rooted in conversational norms. For example, a *more*/*less* asymmetry may arise simply because it seems unnatural or misleading to say that one transgression is less bad than another when both cases are very bad (Grice, 1975). This alternative account predicts that a similar asymmetry may arise whenever people are asked to compare two targets that are both high on the relevant attribute, regardless of whether the judgment is morally relevant. Study 4 casts some doubt on this possibility by demonstrating that the asymmetry is smaller for *would* than *should* judgments, but these two judgment types may differ in other ways. Altogether, considering the limitations of each paradigm, our reluctance-to-downplay account offers a common explanation for both sets of results.

According to our account, people resist scaling down condemnation because—unlike scaling up—it can be construed as downplaying, a signal of bad moral character. Consistent with this account, we found that scaling down can have negative reputational consequences even in contexts in which scaling up offers reputational benefits. Although this evidence suggests that the asymmetry is reinforced by social incentives, we do not necessarily assume that it is solely driven by a desire to signal to others. People may avoid downplaying bad acts not only to avoid looking immoral but also to avoid feeling immoral, even when reputation is not on the line. Of course, public and private signaling concerns can be difficult to disentangle, especially because people may follow a heuristic that reputation is typically at stake. In other words, they may punish and condemn transgressions as if others are watching even when unobserved (Jordan & Rand, 2020).

A reluctance to downplay bad acts may shed light on broader trends in moral condemnation, offering several directions for future research. First, it may contribute to “concept creep,” the tendency for harm-related concepts to broaden over time (Haslam, 2016; Haslam et al., 2020). For instance, when considering whether verbal aggression counts as abuse, people may fear that excluding it from the category will be interpreted as saying that it is not very bad. A similar psychology may also partially explain why initial accusations of wrongdoing can escalate into widespread outrage (Crockett, 2017; Jordan & Kteily, 2023). When discussing a transgression with a group, for instance, people may “want to appear at least as appalled as others” (Sunstein, 2020), and thus they may adjust asymmetrically relative to others’ judgments. If each individual errs toward expressing more (vs. less) condemnation than others, a given transgression may be judged more and more harshly as it is discussed by more people (Schkade et al., 2000).

Of course, moral condemnation is not the only domain in which people are motivated to show sufficient concern. According to our theory, a directional asymmetry may arise for any comparative judgment that implicates moral character (or reputation more broadly), provided that scaling up and scaling down carry different signals. Aside from condemnation of bad acts, future research could examine praise for good acts. When comparing actors who sacrificed different amounts for a good cause, for example, observers might be more willing to say that one person deserves more praise than to say that the other person deserves less—especially if the latter comparison feels like downplaying the actor’s selflessness or the importance of the cause. A similar psychology might also apply to expressions of sympathy (e.g., for natural disasters with different numbers of casualties) or gratitude (e.g., for gifts that cost different amounts of money).

## Conclusion

A straightforward way to determine how much condemnation a moral transgression deserves is to compare it to other transgressions. However, this research demonstrates that the direction of comparison matters. To avoid expressing insufficient condemnation, people scale up more than they scale down.

## Supplemental Material

sj-pdf-1-pss-10.1177_09567976251314972 – Supplemental material for Reluctance to Downplay: Asymmetric Sensitivity to Differences in the Severity of Moral Transgressions Supplemental material, sj-pdf-1-pss-10.1177_09567976251314972 for Reluctance to Downplay: Asymmetric Sensitivity to Differences in the Severity of Moral Transgressions by Amanda E. Geiser, Ike Silver and Deborah A. Small in Psychological Science

## Transparency

*Action Editor:* Yoel Inbar

*Editor:* Patricia J. Bauer

*Author Contributions*

**Amanda E. Geiser:** Conceptualization; Data curation; Formal analysis; Investigation; Methodology; Project administration; Software; Visualization; Writing – original draft.

**Ike Silver:** Conceptualization; Methodology; Project administration; Supervision; Visualization; Writing – review & editing.

**Deborah A. Small:** Conceptualization; Funding acquisition; Methodology; Project administration; Supervision; Visualization; Writing – review & editing.

**Artificial Intelligence:** No artificial-intelligence-assisted technologies were used in this research or the creation of this article.

**Ethics:** This research received approval from the institutional review boards of the University of Pennsylvania and Yale University.

**Open Practices:** All preregistrations, materials, data, and code are publicly available and posted on ResearchBox (https://researchbox.org/928&PEER_REVIEW_passcode=ANAFYW).

## Notes

1. This study was conducted in July 2021, just after Prolific was referenced in a viral TikTok video and received an influx of young female participants (Charalambides, 2021).

2. Here, we use “Celebrity A” for the less severe case and “Celebrity B” for the more severe case.
