# Supplementary material for: *Reluctance to Downplay: Asymmetric Sensitivity to Differences in the Severity of Moral Transgressions
Source: Psychol Sci. 2025 Mar 19;36(3):184–203. doi: 10.1177/09567976251314972 (PMC13428860; doi:10.1177/09567976251314972)
Supplement: sj-pdf-1-pss-10.1177_09567976251314972 – Supplemental material for Reluctance to Downplay: Asymmetric Sensitivity to Differences in the Severity of Moral Transgressions [file sj-pdf-1-pss-10.1177_09567976251314972.pdf]

Supplemental Material

Reluctance to Downplay:

Asymmetric Sensitivity to Differences in the Severity of Moral Transgressions

Table of Contents

SUPPLEMENTAL STUDY 1 .... **2**
SUPPLEMENTAL STUDY 2 .... **10**
STUDY 4 POST-TEST **19**
TRANSGRESSION DESCRIPTIONS FOR ALL STUDIES **21**
TESTS OF RATIO-BASED ADJUSTMENT WITH EXCLUSIONS **25**
TESTS OF ASYMMETRIC ADJUSTMENT WITHIN EACH TRANSGRESSION TYPE **26**
TESTS OF ASYMMETRIC ADJUSTMENT BETWEEN TRANSGRESSION TYPES **32**
TESTS OF ASYMMETRIC ADJUSTMENT OF MORAL OUTRAGE **35**
ORDER EFFECTS ON LESS SEVERE AND MORE SEVERE CASES INDIVIDUALLY **37**
MODERATION ANALYSES FOR STUDIES 2A–2B AND SUPPLEMENTAL STUDY 1 .... **39**
ADDITIONAL ANALYSES FOR STUDY 5 .... **47**
DEVIATIONS FROM PREREGISTRATIONS **49**
REFERENCES **50**

Supplemental Study 1

Supplemental Study 1 was an earlier version of Study 2b, which tested for an order-based asymmetry in three pairs of qualitatively different harms. This study used the same sexual assault stimuli as Study 2b, but different robbery and aggravated assault stimuli. Whereas Study 2b manipulated the severity of robbery and aggravated assault by simply describing the details of each case (e.g., "… leaving him with cuts on his hands and arms" vs. "… leaving him with stab wounds in his chest and abdomen"), Supplemental Study 1 included language that more explicitly contrasted the two cases (e.g., "… left him with severe but non-life-threatening injuries" vs. "… left him with severe and life-threatening injuries"). Although the results of Study 2b suggest that our key results do not hinge on the inclusion of such language, we chose to report the better-controlled version of the study in the main manuscript while reporting the earlier version here as a conceptual replication.

Method

Participants and Design

We requested 600 participants from Prolific and received 600 complete submissions. As preregistered, we excluded submissions from participants who opened the survey more than once under the same participant ID or IP address (n = 15). The final sample consisted of 585 participants (46.7% men, 51.6% women, 1.7% other identity; mean age = 36.0 years). Participants were randomly assigned to one of two *order* conditions: less-severe-first or more-severe-first.

Procedure

As in Study 2b, participants sequentially evaluated three pairs of transgressions, each of which included a less severe case and a more severe case (six cases in total): one pair of *sexual*

assault cases (sexual assault of an adult vs. a child), one pair of *robbery* cases (unarmed vs. armed robbery of a convenience store), and one pair of *aggravated assault* cases (punching vs. attacking one person with a knife). Note that whereas the sexual assault cases used in this study are the same as in Study 2b, the robbery and aggravated assault cases are different. As in Study 2b, the three pairs were presented in random order, but the two cases within a given pair were always presented one after another (one per page). Participants in the less-severe-first condition always evaluated the less severe case before the more severe case within a pair, whereas those in the more-severe-first always evaluated the more severe case before the less severe case.

For each case, participants first assigned a punishment length: "What do you think is an appropriate prison sentence for this offense? ___ years." They entered responses in an open-ended text box that permitted numeric values greater than zero. They also responded to the same two-item punishment scale used in Studies 2a–2b ($r = .95$): "How much should [Target] be punished?" (1 = *No punishment*, 11 = *Most extreme punishment allowable by law*) and "How severe a punishment should be given for this offense?" (1 = *Not at all*, 11 = *Extremely*).

We also included two sets of exploratory measures. On the same page as the punishment measures, participants indicated their moral outrage about a case: "How morally outraged were you by this offense?" (1 = *Not at all*, 11 = *Extremely*). On a separate page at the end of the survey, they rated their moral conviction (Skitka & Morgan, 2014) about each category of transgressions (e.g., aggravated assault), without reference to the more or less severe cases evaluated earlier. For each issue, participants indicated to what extent their position on the issue is "a reflection of your core moral beliefs and convictions" and "connected to your beliefs about fundamental right and wrong" (1 = *Not at all*, 5 = *Very much*).

Results

To assess the amount that participants adjusted condemnation from one case to another, we created two within-participant difference scores (one for each punishment measure). That is, for each participant and for each measure, we subtracted their punishment of the less severe case from their punishment the more severe case. For the open-ended measure of punishment length (i.e., number of years in prison), we also created a ratio-based measure of adjustment by dividing the punishment length assigned to the more severe case by the punishment length assigned to the less severe case.

Prison Sentences

We began by testing for an asymmetry in the amount that participants adjusted punishment length from one case to the next. We preregistered excluding 5% of observations associated with unrealistically high punishment lengths. As specified in our preregistration, we flagged the highest 5% of responses for the more severe case, out of the 1,755 responses across all participants and all three transgression categories. The participant-scenario observations that were flagged were excluded from analyses of absolute adjustment as well as from analyses of punishment length for each case individually. In total, 85 out of 1,755 observations were flagged for exclusion (assigning punishments that ranged from 65 years to 1,000,000 years).

We conducted a linear regression with adjustment of punishment length predicted by order (-0.5 = less-severe-first, +0.5 = more-severe-first), including fixed effects for each transgression type. We clustered standard errors by participant to account for the fact that each participant provided up to three observations. We observed a significant effect of order on adjustment between cases. Overall, participants who started with the less severe case adjusted by an average of 7.69 (SD = 8.79) years, whereas those who started with the more severe case first adjusted only 5.14 (SD = 7.55) years, b = -2.49, clustered SE = 0.45, t = -5.56, p < .001. This

effect was only significant for the pair of sexual assault cases, $t = -9.97$, $p < .001$. It was non-significant for the pair of robbery cases, $t = -0.71$, $p = .477$, and the pair of aggravated assault cases, $t = -0.73$, $p = .465$. Table S1 shows the punishment lengths assigned to each individual case and adjustment between cases.

Participants who started with the less severe case assigned it an average of 7.19 (SD = 6.95) years of punishment, while those who started with the more severe case assigned it 10.61 (SD = 10.23) years, $b = 3.37$, clustered $SE = 0.49$, $t = 6.82$, $p < .001$. In contrast, order of evaluation did not have a significant effect on the length of punishment assigned to the more severe case. Participants who started with the less severe case assigned it an average of 14.88 (SD = 12.80) years of prison time, while those who started with the more severe case assigned it an average of 15.75 (SD = 12.33) years, $b = 0.88$, clustered $SE = 0.78$, $t = 1.12$, $p = .261$.

The same order-based asymmetry that we found in absolute adjustment also emerged for ratio-based adjustment, to which we did not apply any exclusions. Participants who started with the less severe case assigned a punishment to the more severe case that was 2.78 times as long (SD = 2.94) as the less severe case, whereas those who started with the more severe case assigned it a punishment that was only 2.15 times as long (SD = 2.49), $b = 0.63$, clustered SE = 0.13, $t = 4.96$, $p < .001$ $b = 0.63$, clustered $SE = 0.13$, $t = 4.96$, $p < .001$.

We also explored the frequency with which participants refrained from adjusting at all (i.e., assigned equal punishment lengths to both cases). Most participants who started with the less severe case adjusted by some amount; however, those who started with the more severe case assigned equal punishment more frequently. Participants who evaluated the less severe case first refrained from adjusting 10.0% of the time, whereas those who evaluated the more severe case first did so 27.2% of the time, $b = 1.39$, $OR = 4.02$, clustered $SE = 0.18$, $z = 7.76$, $p < .001$.

Table S1
Mean Punishment Length for Each Case and Adjustment Between Cases in Supplemental Study 1

	Sexual Assault		Robbery		Aggravated Assault	
	Less Severe First	More Severe First	Less Severe First	More Severe First	Less Severe First	More Severe First
Less Severe Case	11.58 (8.71)	17.73*** (12.55)	4.65 (4.24)	5.76** (5.34)	5.78 (5.32)	8.91*** (7.67)
More Severe Case	20.41 (14.80)	19.57 (13.42)	9.22 (7.30)	10.03 (8.33)	15.58 (12.85)	18.12* (12.62)
Absolute Adjustment	8.82 (9.87)	1.85*** (5.67)	4.56 (4.64)	4.28 (5.04)	9.80 (10.00)	9.21 (9.31)
Ratio-Based Adjustment	2.12 (1.79)	1.22*** (0.76)	2.63 (3.43)	2.03** (1.24)	3.57 (3.15)	3.19 (3.82)
Frequency of Adjustment	84.2%	41.2%***	91.8%	86.7%†	94.2%	90.5%†

Note: Standard deviations are given in parentheses. Indications of statistical significance are based on linear regressions comparing the punishment assigned to a case by participants who evaluated the less severe vs. more severe case first. † $p < .10$, * $p < .05$, ** $p < .01$, *** $p < .001$

Scale-Based Punishment Measure

An order-based asymmetry also emerged in the extent to which participants adjusted on the 11-point scale-based punishment measure. Participants who started with the less severe case adjusted by an average of 1.43 points (*SD* = 1.42), while those who started with the more severe case adjusted by only 1.21 (*SD* = 1.42), $b = 0.22$, clustered *SE* = 0.07, $t = 3.16$, $p = .002$. This effect was significant for the pair of sexual assault cases, $t = -8.15$, $p < .001$, but it was non-significant for the pair of robbery cases, $t = -0.53$, $p = .594$, and non-significant for the pair of aggravated assault cases, $t = 0.27$, $p = .790$ (Table S2). Overall, order did not have a significant effect on scale-based punishment of either the less severe or more severe case individually, $|t|$s < 1.

Participants who started with the less severe case assigned equal punishment 22.5% of the time, whereas those who started with the more severe case did so 30.5% of the time, $b = 0.48$, $OR = 1.61$, clustered $SE = 0.14$, $z = 3.51$, $p < .001$.

Note that these results are an especially valuable complement to the results of the open-ended measure, given that the open-ended measure was bounded only on the lower end while the scale-based measure was bounded on both ends. On the open-ended measure, participants essentially always had more space to adjust upward than downward, thus biasing results in favor of our predicted asymmetry. In contrast, the scale-based measure provided a more conservative test: If a participant started above the midpoint of the scale (which held true in 77% of observations), they would have had more space to adjust downward than upward, thus biasing results *against* our predicted asymmetry.

Table S2
Mean Scale-Based Punishment (1-11) for Each Pair of Transgressions and Adjustment Between Cases in Supplemental Study 1

	Sexual Assault		Robbery		Aggravated Assault	
	Less Severe First	More Severe First	Less Severe First	More Severe First	Less Severe First	More Severe First
Less Severe Case	8.95 (1.42)	9.24* (1.63)	5.95 (1.81)	5.80 (1.96)	7.18 (1.42)	7.36 (1.63)
More Severe Case	9.83 (1.82)	9.50** (1.57)	7.56 (2.16)	7.35 (2.21)	8.98 (2.20)	9.19† (2.03)
Absolute Adjustment	0.89 (1.13)	0.25*** (0.70)	1.61 (1.39)	1.55 (1.28)	1.80 (1.55)	1.83 (1.57)
Frequency of Non-Adjustment	34.7%	61.9%***	15.8%	14.3%	16.8%	15.3%

Note: Standard deviations are given in parentheses. Indications of statistical significance are based on linear regressions with the amount participants adjusted the length of punishment between the two cases (more severe minus less severe) predicted by order of evaluation (-0.5 = less-severe-first, +0.5 = more-severe-first). † p < .10, * p < .05, ** p < .01, *** p < .001

Adjustment Between Transgression Types

We also explored how much participants adjusted *between* the different pairs of transgressions as a function of the random order in which they appeared. Table S3 shows the average amounts that participants adjusted on each measure between pairs.

For the open-ended punishment length measure, we applied the same exclusion rule used in our main analyses. We conducted a linear regression with adjustment of punishment length predicted by category order (-0.5 = less-severe-category-first, +0.5 = more-severe-category-first), with fixed effects for each pair of categories and with standard errors clustered by participant. Overall, we observed a between-category asymmetry similar to the one we observed within transgression pairs. Participants who started with the less severe category adjusted punishment length by an average of 8.73 (SD = 10.92) years between categories, while those who started with the more severe category adjusted by an average of 7.25 (SD = 11.41) years, b = -1.73, clustered SE = 0.50, t = -3.45, p < .001.

We observed a similar pattern on the scale-based punishment measure. Participants who started with the less severe category adjusted by an average of 1.94 (SD = 1.80) scale points, while those who started with the more severe category adjusted by an average of 1.58 (SD = 1.76) points, b = -0.43, clustered SE = 0.07, t = -6.11, p < .001.

Table S3
Adjustment of Punishment Between Pairs of Transgressions in Supplemental Study 1

	Robbery (Less Severe) vs. Aggravated Assault (More Severe)		Robbery (Less Severe) vs. Sexual Assault (More Severe)		Aggravated Assault (Less Severe) vs. Sexual Assault (More Severe)	
	Less Severe Category First	More Severe Category First	Less Severe Category First	More Severe Category First	Less Severe Category First	More Severe Category First
Adjustment of Average Punishment Length	5.53 (7.67)	5.06 (8.61)	12.81 (12.30)	11.61 (12.11)	8.45 (11.36)	4.88** (12.00)
Adjustment of Average Scale-Based Punishment	1.46 (1.46)	1.27 (1.49)	3.06 (1.93)	2.53 (1.92)	1.44 (1.53)	0.87 (1.35)

Note: Standard deviations are given in parentheses. Punishment length for a given pair was the average punishment length assigned to the less severe case and the more severe case within that pair. Indications of statistical significance are based on linear regressions with the amount participants adjusted the length of punishment between two pairs (more severe minus less severe) predicted by order of evaluation (-0.5 = less-severe-category-first, +0.5 = more-severe-category-first). The ranking that we used for the relative severity of each transgression type was: sexual assault, aggravated assault, robbery. † $p < .10$, * $p < .05$, ** $p < .01$, *** $p < .001$

Supplemental Study 2

Supplemental Study 2 tested whether the order-based asymmetry is attenuated for less harmful pairs of transgressions. If actions that are perceived as less harmful seem less important to condemn, as we found in Study 5, then people may not be as reluctant to downplay transgressions that caused very little harm. As a result, they may adjust condemnation asymmetrically when sequentially evaluating a pair of serious harms (e.g., deaths) but adjust more symmetrically when evaluating a pair of more minor harms (e.g., headaches), even if the perpetrators' actions themselves are held constant (e.g., nurses failing to give patients their medication). Supplemental Study 2 tested for this possibility.

Method

Participants and Design

We requested 800 participants from Prolific and received 799 complete responses. After excluding participants who opened the survey more than once under the same participant ID or IP address ($n = 4$), the final sample consisted of 795 participants (50.1% men, 47.9% women, 2.0% other identity; mean age = 38.4 years). Participants were assigned to one of eight conditions in a 2 (order: less-severe-first vs. more-severe-first) X 2 (harm: high vs. low) X 2 (scenario: nurse vs. driver) full-factorial design.

Procedure

The procedure was similar to that of Studies 1–2b and Supplemental Study 1. Participants sequentially evaluated a pair of transgressions in which one case was transparently more severe than the other (based on a quantitative indicator of severity). Some participants considered the less severe case first, whereas others considered the more severe case first.

We included two different scenarios to explore robustness across different contexts. Participants assigned to the nurse scenario considered two cases in which nurses skipped their shifts and missed giving patients their medication. In the high-harm condition, this was a heart medication; one nurse caused two patients to die (less severe case), and the other nurse caused four patients to die (more severe case). In the low-harm condition, the medication was for headaches; one nurse caused two patients to experience headaches for a few hours (less severe case), and the other nurse caused four patients to experience headaches for a few hours (more severe case). Participants assigned to the driver scenario instead considered two cases in which a driver was texting and swerved off the road. In the high-harm condition, one driver struck and killed one pedestrian (less severe case), and the other struck and killed three pedestrians (more severe case). In the low-harm condition, one driver struck and damaged one parked car (less severe case), and the other struck and damaged three parked cars (more severe case).

For each case, participants first assigned a punishment length: “For how long (if at all) should this person's [nursing or driver’s] license be revoked as a result of their actions? ___ years.” They entered responses in an open-ended text box that permitted numeric values greater than or equal to zero, given that we did not explicitly specify that any target would be punished. Participants also indicated how much punishment they thought each target deserved on a scale from 0 (*No punishment*) to 10 (*The death penalty*). Each case was presented on a separate page.

Results

To assess the amount that participants adjusted condemnation from one case to another, we used the same procedure as Study 1 and Studies 2a–2b to create two within-participant difference scores (one for each punishment measure). That is, for each participant and for each measure, we subtracted their punishment of the less severe case from their punishment the more

severe case. For the scale-based punishment measure, we also created a binary variable indicating whether a participant assigned equal punishment to both cases (1 = yes, 0 = no).

Punishment Lengths

We began by testing for an asymmetry in the absolute amount that participants adjusted punishment length from one case to the next. As in Studies 2a–2b, we preregistered excluding 5% of observations associated with unrealistically high responses for the more severe case. We flagged the highest 5% of responses for the more severe case out of the 795 responses across all participants in all conditions. The 10 participants whose responses were flagged were excluded from analyses of punishment length adjustment (these participants assigned punishments to the more severe case ranging from 120 to 10,000 years).

We conducted a linear regression with punishment length adjustment predicted by order (-0.5 = less-severe-first, +0.5 = more-severe-first), harm (-0.5 = low, +0.5 = high), and the two-way interaction between these variables, including fixed effects for each scenario. We observed a significant main effect of order, in line with the asymmetry observed in previous studies: Overall, participants who started with the less severe case adjusted more than those who started with the more severe case, $b = -2.27$, $SE = 0.81$, $t(780) = -2.81$, $p = .005$. There was also a significant main effect of harm, such that participants adjusted more within pairs of high-harm cases than within pairs of low-harm cases, $b = 6.90$, $SE = 0.81$, $t(780) = 8.55$, $p < .001$. Of particular interest, we observed a significant two-way interaction, $b = 4.89$, $SE = 1.61$, $t(780) = 3.03$, $p = .003$. To unpack this interaction, we examined the simple effect of order separately for participants in the high-harm and low-harm conditions.

When considering a pair of more harmful transgressions, participants who started with the less severe case adjusted between the two cases by a larger amount ($M = 9.66$ years, $SD =$

18.54) than those who started with the more severe case (*M* = 4.96 years, *SD* = 13.14), *b* = -4.72, *SE* = 1.15, *t*(780) = -4.12, *p* < .001. By contrast, when considering a pair of less harmful transgressions, participants who started with the less severe case adjusted no more (*M* = 0.31 years, *SD* = 2.45) than those who started with the more severe case (*M* = 0.46 years, *SD* = 1.41), *b* = -0.17, *SE* = 1.14, *t*(780) = -0.15, *p* = .878.

In addition to the analysis of absolute adjustment of punishment length, we also examined the ratio between punishment lengths assigned to the more severe versus less severe case. For this analysis, we excluded observations from participants who assigned 0 years of punishment for the less severe case (otherwise, the ratio would be undefined). We also applied the exclusion criterion that we used to remove extreme responses from analyses of absolute adjustment. As we observed in absolute adjustment, participants who started with the less severe case adjusted more than those who started with the more severe case, *b* = 0.27, *SE* = 0.08, *t* = 3.30, *p* = .001. Those considering a pair of actions that caused greater harm adjusted more than those considering pairs of actions that caused less harm, *b* = 0.26, *SE* = 0.08, *t* = 3.12, *p* = .002. Importantly, the interaction between order and harm was significant, *b* = 0.48, *SE* = 0.16, *t* = 2.96, *p* = .003. When considering a pair of more serious harms, participants who started with the less severe case assigned the more severe case a punishment 1.84 times as long as the less severe case on average (*SD* = 1.44), whereas those who started with the more severe case assigned the more severe case a punishment 1.33 times as long (*SD* = 0.84), *b* = 0.51, *SE* = 0.10, *t* = 4.96, *p* < .001. Meanwhile, when considering a pair of less serious harms, participants who started with the less severe case adjusted no more (*M* = 1.41, *SD* = 0.64) than those who started with the more severe case (*M* = 1.37, *SD* = 0.74), *b* = 0.03, *SE* = 0.13, *t* = 0.22, *p* = .83.

We also explored whether participants adjusted on the open-ended punishment length measure at all by conducting a series of logistic regressions. Overall, participants refrained from adjusting more frequently when they started with the more severe case than when they started with the less severe case, $b = 0.50$, $OR = 1.65$, $SE = 0.16$, $z = 3.12$, $p = .002$. Participants considering pairs of more outrageous harms adjusted less frequently than those considering pairs of less outrageous harms, $b = 0.96$, $OR = 0.38$, $SE = 0.03$, $z = -5.90$, $p < .001$. Of particular interest, the interaction between order and harm was significant, $b = 0.87$, $OR = 2.38$, $SE = 0.32$, $z = 2.68$, $p = .007$. When considering a pair of more serious harms, 40.0% of participants who started with the less severe case refrained from differentiating between cases, whereas 72.5% of those who started with the more severe case refrained from differentiating, $b = 0.94$, $OR = 2.55$, $SE = 0.22$, $z = 4.31$, $p < .001$. By contrast, when considering a pair of less serious harms, participants who started with the less severe case refrained from differentiating between cases no more or less frequently (55.6%) than those who started with the more severe case (62.9%), $b = 0.07$, $OR = 1.07$, $SE = 0.24$, $z = 0.29$, $p = .770$.

Punishment Ratings

A similar pattern of results emerged on the scale-based punishment measure. Overall, participants adjusted more when starting with the less severe case than when starting with the more severe case, $b = -0.21$, $SE = 0.06$, $t(790) = -3.54$, $p < .001$. They also adjusted somewhat more between high-harm transgressions than between low-harm transgressions, $b = 0.15$, $SE = 0.06$, $t(790) = 2.60$, $p = .010$. However, these two main effects were again qualified by a significant two-way interaction, $b = -0.55$, $SE = 0.12$, $t(790) = 4.64$, $p < .001$.

When considering a pair of more harmful transgressions, participants who started with the less severe case adjusted more ($M = 0.84$, $SD = 0.90$) than those who started with the more

severe case (*M* = 0.36, *SD* = 0.80), *b* = -0.48, *SE* = 0.08, *t*(790) = -5.80, *p* < .001. But when considering a pair of less harmful transgressions, participants adjusted no more if they started with the less severe case (*M* = 0.41, *SD* = 0.73) than if they started with the more severe case (*M* = 0.47, *SD* = 0.93), *b* = 0.07, *SE* = 0.08, *t*(790) = 0.78, *p* = .437.

Overall, participants also refrained from differentiating between cases on the scale-based punishment measure more frequently when starting with the more severe case than when starting with the less severe case, *b* = 0.20, *SE* = 0.03, *t*(790) = 5.99, *p* < .001. Participants considering a pair of more harmful transgressions did not adjust more or less frequently than those considering a pair of less harmful transgressions, *b* = 0.03, *SE* = 0.03, *t*(790) = 0.88, *p* = .381. However, we again observed a significant interaction between order and harm, *b* = 0.25, *SE* = 0.07, *t*(790) = 3.82, *p* < .001. When considering a pair of more serious harms, 40.0% of participants who started with the less severe case assigned equal punishment to both cases, whereas 72.5% of those who started with the more severe case did so, *b* = 0.33, *SE* = 0.05, *t*(790) = 6.96, *p* < .001. But when considering a pair of less serious harms, participants who started with the less severe case assigned equal punishment to both cases no more or less frequently (55.6%) than those who started with the more severe case (62.9%), *b* = 0.07, *SE* = 0.05, *t*(790) = 1.53, *p* = .127.

Note that for this study in particular, the scale-based punishment measure provided an especially conservative test of our predictions. If participants start above the scale midpoint, as did 72.2% of participants in the high-harm condition, results should be biased against our proposed asymmetry. But if participants start below the scale midpoint, as did 71.6% of participants in the low-harm condition, results should be biased *in favor of* this asymmetry. However, we consistently found the opposite: The predicted asymmetry emerged for pairs of more harmful transgressions, but did not emerge at all for pairs of less harmful transgressions.

Order Effects on Less Severe Case

There was a significant main effect of order on the length of punishment assigned to the less severe case, $b = 3.97$, $SE = 1.63$, $t(780) = 2.44$, $p = .015$, such that participants assigned significantly longer punishments to the less severe case when they evaluated it after (vs. before) the more severe case. Unsurprisingly, the less severe case received a longer punishment when it caused high (vs. low) harm, $b = 25.99$, $SE = 1.63$, $t(780) = 15.99$, $p < .001$. There was also a significant interaction between order and harm, $b = 8.38$, $SE = 3.25$, $t(780) = 2.58$, $p = .010$. For pairs of high-harm transgressions, the less severe case received a significantly longer punishment if it was evaluated second (vs. first), $b = 8.16$, $SE = 2.31$, $t(780) = 3.54$, $p < .001$. But for the pairs of low-harm transgressions, punishment of the less severe case was not influenced by order, $t < 1$. Table S4 shows order effects on punishment length for each case.

On the scale-based punishment measure, we observed a marginally significant main effect of order, $b = 0.24$, $SE = 0.14$, $t(780) = 1.77$, $p = .078$, such that the less severe transgression received more punishment when it was evaluated second rather than first. Again, there was a significant main effect of harm, $b = 3.00$, $SE = 0.14$, $t(780) = 22.07$, $p < .001$. However, the interaction between order and harm was not significant, $b = 0.12$, $SE = 0.27$, $t(780) = 0.43$, $p = .668$. See Table S5 for scale-based punishment judgments for each case.

Order Effects on More Severe Case

When examining the length of punishment assigned to the more severe case, the main effect of order was not significant, $b = 1.69$, $SE = 1.81$, $t(780) = 0.94$, $p = .349$. The more severe case received a longer punishment when it caused high (vs. low) harm, $b = 32.89$, $SE = 1.81$, $t(780) = 18.18$, $p < .001$. The interaction between order and harm was non-significant, $b = 3.49$,

SE = 3.62, *t*(780) = 0.97, *p* = .335. See Table S4 for punishment lengths assigned to the more severe case within each individual pair of transgressions.

A similar pattern emerged in scale-based punishment judgments: The main effect of order was not significant, *b* = 0.03, *SE* = 0.14, *t*(780) = 0.23, *p* = .821, and the main effect of harm was significant, *b* = 3.15, *SE* = 0.14, *t*(780) = 22.98, *p* < .001. The interaction between order and harm was also non-significant, *b* = -0.43, *SE* = 0.27, *t*(780) = -1.57, *p* = .116. See Table S5 for scale-based punishment judgments of the more severe case within each pair of transgressions.

Table S4
Mean Punishment Length for Each Case in Supplemental Study 2

		Nurse Scenario		Driver Scenario	
		Less Severe First	More Severe First	Less Severe First	More Severe First
High-Harm Version	Less Severe Case	24.95 (33.30)	38.09* (38.91)	21.79 (26.03)	25.21 (28.00)
	More Severe Case	30.73 (36.83)	40.64† (39.67)	35.22 (32.19)	32.47 (33.69)
Low-Harm Version	Less Severe Case	0.74 (2.31)	0.72 (1.64)	2.36 (6.93)	2.00 (2.26)
	More Severe Case	0.99 (3.45)	1.03 (2.74)	2.73 (5.44)	2.61 (2.99)

Note: Standard deviations are given in parentheses. Indications of statistical significance are based on linear regressions comparing the punishment assigned to a case by participants who evaluated the less severe vs. more severe case first. † p < .10, * p < .05, ** p < .01, *** p < .001

Table S5
Mean Scale-Based Punishment Rating for Each Case in Supplemental Study 2

		Nurse Scenario		Driver Scenario	
		Less Severe First	More Severe First	Less Severe First	More Severe First
High-Harm Version	Less Severe Case	5.16 (2.43)	5.78† (2.27)	6.70 (1.40)	6.69 (1.59)
	More Severe Case	5.85 (2.54)	5.97 (2.24)	7.68 (1.37)	7.20* (1.42)
Low-Harm Version	Less Severe Case	2.49 (1.60)	2.52 (2.00)	3.49 (1.94)	3.83 (1.88)
	More Severe Case	2.72 (1.65)	2.89 (1.99)	4.07 (2.02)	4.40 (1.95)

Note: Standard deviations are given in parentheses. Indications of statistical significance are based on linear regressions comparing the punishment assigned to a case by participants who evaluated the less severe vs. more severe case first. † $p < .10$, * $p < .05$, ** $p < .01$, *** $p < .001$

Study 4 Post-Test

In Study 4, we tested whether the *more*/*less* asymmetry is more pronounced when judging how much punishment a pair of transgressions *should* receive than when judging how much punishment those transgressions *would* receive. Our assumption was that *should* judgments may yield a larger asymmetry because they implicate moral character more strongly than *would* judgments. We conducted a post-test to test this assumption.

Method

Participants and Design

We requested 400 participants from Prolific and received 400 complete submissions. After excluding participants who opened the survey more than once under the same participant ID or IP address (n = 7), the final sample consisted of 393 participants (48.9% men, 49.6% women, 1.5% other identity; mean age = 37.4 years). Participants were randomly assigned to either the *should* or *would* condition.

Procedure

Participants were asked to imagine being presented with a pair of criminal offenses, both of which are punishable by a prison sentence. Those in the *should* condition were then asked to imagine making a judgment about "how much punishment each case should receive based on YOUR PERSONAL FEELINGS." Meanwhile, those in the *would* condition were asked to imagine making a judgment about "how much punishment each case would receive in a COURT OF LAW." Participants were then asked: "To what extent would your answer say something about your moral character?" (0 = *Not at all*, 10 = *Very much*).

Results

In line with our assumption that people believe *should* judgments more strongly implicate moral character than *would* judgments, participants indicated that a judgment of how much punishment a pair of criminal offenses *should* receive would say more about their moral character (M = 8.03, SD = 1.84) than a judgment of how much punishment a pair of criminal offenses *would* receive (M = 7.01, SD = 2.63), $t(391) = 4.44$, $p < .001$.

Transgression Descriptions For all Studies

Table S6: Description of Each Pair of Transgressions Used in Each Study

Study	*Transgression Category*	*Difference in Severity*	*Less Severe Case*	*More Severe Case*
**Study 1**	Sexual misconduct	Both quantitative and qualitative	"Louis CK, a stand-up comedian and filmmaker, was accused of sexual misconduct by five women in the fall of 2017. Many of the allegations involved CK pressuring unwilling women — often aspiring female comedians looking to connect with CK professionally — to watch him masturbate. He did not physically assault any of these women."	"Harvey Weinstein, an award-winning film producer, was accused of rape and sexual abuse by more than 80 women in the fall of 2017. Many of the cases, which spanned a period of more than 30 years, involved Weinstein inviting young actresses or models to his hotel room to discuss their careers and then pressuring them to have sex with him."
**Study 2a**	Sexual assault	Quantitative	"Alex sexually assaulted two young girls in a public restroom. He was found guilty of sexual assault."	"Harry sexually assaulted three young girls in a public restroom. He was found guilty of sexual assault."
	Robbery	Quantitative	"Alex robbed a convenience store armed with a gun and left with thousands of dollars. He was found guilty of robbery."	"Harry robbed several convenience stores armed with a gun and left with thousands of dollars. He was found guilty of robbery."
	Aggravated assault	Quantitative	"Alex attacked an elderly man with a knife and left him with severe and life-threatening injuries. He was found guilty of aggravated assault."	"Harry attacked an elderly man and an elderly woman with a knife and left them with severe and life-threatening injuries. He was found guilty of aggravated assault."
**Study 2b**	Sexual assault	Qualitative	"Alex sexually assaulted an adult woman in a public restroom. Alex was found guilty of sexual assault."	"Harry sexually assaulted a young girl in a public restroom. Harry was found guilty of sexual assault."
	Robbery	Qualitative	"Alex robbed a convenience store, raised his fist at the cashier, and threatened to hurt her if she didn't hand over all the money in the register. Alex was found guilty of robbery."	"Harry robbed a convenience store, pointed a gun at the cashier, and threatened to kill her if she didn't hand over all the money in the register. Harry was found guilty of robbery."
	Aggravated assault	Qualitative	"Alex attacked an elderly man with a knife, leaving him with cuts on his hands and arms. Alex was found guilty of aggravated assault."	"Harry attacked an elderly man with a knife, leaving him with stab wounds in his chest and abdomen. Harry was found guilty of aggravated assault."

	Aggravated assault	Quantitative	"Person A attacked an elderly man with a knife. He was found guilty of aggravated assault."	"Person B attacked an elderly man and an elderly woman with a knife. He was found guilty of aggravated assault."
	Kidnapping	Quantitative	"Person A kidnapped two young children who were unaccompanied by adults in public areas. He was found guilty of kidnapping."	"Person B kidnapped four young children who were unaccompanied by adults in public areas. He was found guilty of kidnapping."
**Study 3**	Manslaughter	Quantitative	"Person A struck and killed one person while drunk driving. He was found guilty of manslaughter."	"Person B struck and killed three people while drunk driving. He was found guilty of manslaughter."
	Robbery	Quantitative	"Person A robbed one convenience store armed with a gun and stole thousands of dollars. He was found guilty of robbery."	"Person B robbed several convenience stores armed with a gun and stole tens of thousands of dollars. He was found guilty of robbery."
	Sexual assault	Qualitative	"Person A sexually assaulted an adult woman in a public restroom. He was found guilty of sexual assault."	"Person B sexually assaulted a young girl in a public restroom. He was found guilty of sexual assault."
**Study 4**	Manslaughter	Quantitative	"Person A struck and killed one pedestrian while driving under the influence of alcohol.<br><br>Person A was found guilty of one count of manslaughter."	"Person B struck and killed three pedestrians while driving under the influence of alcohol.<br><br>Person B was found guilty of three counts of manslaughter."
	School shooting	Quantitative	Person A planned and carried out a school shooting, resulting in the death of one child.	Person B planned and carried out a school shooting, resulting in the death of three children.
	Sexual assault	Quantitative	Person A sexually assaulted one woman in a public restroom.	Person B sexually assaulted three women in a public restroom.
**Study 5**	Knife attack	Quantitative	Person A attacked one person with a knife, leaving them with life-threatening injuries.	Person B attacked three people with a knife, leaving them with life-threatening injuries.
	Vehicular manslaughter	Quantitative	Person A struck and killed one pedestrian while driving under the influence of alcohol.	Person B struck and killed three pedestrians while driving under the influence of alcohol.

	Carjacking	Quantitative	Person A forced one driver out of their vehicle and stole the vehicle.	Person B forced three drivers out of their vehicles and stole the vehicles.
	Burglary	Quantitative	Person A broke into one person's house while the owner was not home.	Person B broke into three people's houses while the owners were not home.
	Online harassment	Quantitative	Person A repeatedly harassed one person on the internet with threatening messages.	Person B repeatedly harassed three people on the internet with threatening messages.
	Mail theft	Quantitative	Person A stole mail from one person's mailbox.	Person B stole mail from three people's mailboxes.
	Prescribing wrong medication	Quantitative	Person A, a medical doctor, prescribed the wrong medication to one patient, resulting in hospitalization.	Person B, a medical doctor, prescribed the wrong medication to three patients, resulting in hospitalization.
	Denting cars	Quantitative	Person A struck and left dents in one person's parked car, and then drove away.	Person B struck and left dents in three people's parked cars, and then drove away.
	Feeding children an allergen	Quantitative	Person A, an elementary school teacher, provided snacks that contain nuts to one student with a severe nut allergy.	Person B, an elementary school teacher, provided snacks that contain nuts to three students with severe nut allergies.
	Bar fighting	Quantitative	Person A punched one person during a fight at a bar, leaving them with bruises.	Person B punched three people during a fight at a bar, leaving them with bruises.
**Study 6**	Sexual assault	Both quantitative and qualitative	Celebrity A was accused of sexually assaulting 4 women between the ages of 29 and 40.	Celebrity B was accused of sexually assaulting 30 girls and women between the ages of 14 and 37.
**Supplemental Study 1**	Sexual assault	Qualitative	"Alex sexually assaulted an adult woman in a public restroom. He was found guilty of sexual assault."	"Harry sexually assaulted a young girl in a public restroom. He was found guilty of sexual assault."
	Robbery	Qualitative	"Alex robbed a convenience store unarmed and left with thousands of dollars. He was found guilty of robbery."	"Harry robbed a convenience store armed with a gun and left with thousands of dollars. He was found guilty of robbery."

	Aggravated assault	Qualitative	"Alex punched an elderly man and left him with severe but non-life-threatening injuries. He was found guilty of aggravated assault."	"Harry attacked an elderly man with a knife and left him with severe and life-threatening injuries. He was found guilty of aggravated assault."
**Supplemental Study 2**	Nurse missing medication (low-harm)	Quantitative	"Person A, a nurse who works with dementia patients, skipped their shift and missed giving two patients their headache medication. As a consequence these two patients experienced headaches for a few hours."	"Person B, a nurse who works with dementia patients, skipped their shift and missed giving four patients their headache medication. As a consequence these four patients experienced headaches for a few hours."
	Nurse missing medication (high-harm)	Quantitative	"Person A, a nurse who works with dementia patients, skipped their shift and missed giving two patients their heart medication. As a consequence these two patients died."	"Person B, a nurse who works with dementia patients, skipped their shift and missed giving four patients their heart medication. As a consequence these four patients died."
	Distracted driving (low-harm)	Quantitative	"Person A was texting while driving and swerved off the road, striking and causing damage to one parked car."	"Person B was texting while driving and swerved off the road, striking and causing damage to three parked cars."
	Distracted driving (high-harm)	Quantitative	"Person A was texting while driving and swerved off the road, striking and killing one pedestrian."	"Person B was texting while driving and swerved off the road, striking and killing three pedestrians."

Note. This table provides the description of each pair of transgressions used across all studies. For each pair, we include the transgression category, whether the two cases in a pair differed in severity quantitatively or qualitatively (or both), and the descriptions of both cases shown to participants. In Studies 1, 2a–2b, and 3–5, participants evaluated all cases shown here. In Supplemental Study 2, each participant evaluated only one of the four pairs, but they always evaluated both cases within the same pair. In Study 1, a screenshot of a newspaper headline and an image of the target appeared below the description of each case (see materials on ResearchBox). The names used in Studies 2a–2b were randomized, and participants saw a different name for each of the six transgressions they evaluated. In Studies 3–5 and Supplemental Study 2, whether a target was described as "Person A" or "Person B" depended on the randomized order in which the two cases appeared (i.e., whichever case was presented first or on the left side of the page described the actions of "Person A" and whichever case was presented second or on the right side of the page described the actions of "Person B"

Tests of Ratio-Based Adjustment with Exclusions

Studies 2a–2b

In our preregistrations for Studies 2a–2b, we stated that we would analyze ratio-based adjustment in prison sentences without applying any exclusions. However, after collecting the data for Study 2b, we realized that our ability to detect an asymmetry in ratio-based adjustment may have been limited by noise from extreme responses. Though we stuck to our preregistered plan (i.e., analyzing the ratio-based adjustment measure without exclusions) in the main text, we also conducted exploratory analyses of ratio-based adjustment after applying the exclusion rule that we used in analyses of absolute adjustment (described in the main text).

In both studies, after applying exclusions, an overall order-based asymmetry emerged in the ratio between prison sentences assigned to the more severe versus less severe case. In Study 2a, the ratio between punishment lengths was smaller on average among participants who started with the more severe case (M = 1.17, SD = 0.39) than those who started with the less severe case (M = 1.42, SD = 0.45), b = -0.25, clustered SE = 0.02, t = -11.13, $p < .001$. This pattern held for each of the three pairs of transgressions: the pair of sexual assault cases, b = -0.23, SE = 0.02, $t(566)$ = -9.60, $p < .001$; the pair of robbery cases, b = -0.30, SE = 0.04, $t(583)$ = -6.68, $p < .001$; and the pair of aggravated assault cases, b = -0.21, SE = 0.03, $t(581)$ = -6.65, $p < .001$.

Likewise, in Study 2b, the ratio between punishment lengths was smaller on average among participants who started with the more severe case (M = 1.71, SD = 1.91) than those who started with the less severe case (M = 2.08, SD = 1.44), b = -0.37, clustered SE = 0.08, t = -4.43, $p < .001$. This effect held for the pair of sexual assault cases, b = -0.64, SE = 0.06, $t(521)$ = -9.94, $p < .001$; and the pair of aggravated assault cases, b = -0.23, SE = 0.07, $t(562)$ = -3.10, p = .002; but was not significant for the pair of robberies, b = -0.27, SE = 0.21, $t(580)$ = -1.26, p = .209.

Tests of Asymmetric Adjustment within Each Transgression Type

Studies 2a–2b

Prison Sentences

In both studies, participants adjusted less between cases on average when starting with the more severe case than when starting with the less severe case. In Study 2a, in which participants evaluated pairs of *quantitatively* different harms, this asymmetry emerged for the pair of sexual assault cases, $b = -5.39$, $SE = 0.67$, $t(566) = -8.00$, $p < .001$; the pair of robbery cases, $b = -3.24$, $SE = 0.44$, $t(583) = -7.42$, $p < .001$; and the pair of aggravated assault cases, $b = -4.02$, $SE = 0.74$, $t(581) = -5.46$, $p < .001$. In Study 2b, in which participants evaluated pairs of *qualitatively* different harms, this effect was significant for the pair of sexual assault cases, $b = -8.12$, $SE = 0.78$, $t(521) = -10.43$, $p < .001$; and the pair of aggravated assault cases, $b = -1.43$, $SE = 0.65$, $t(562) = -2.21$, $p = .028$; but it was not significant for the pair of robbery cases, $b = -0.88$, $SE = 0.75$, $t(580) = -1.18$, $p = .240$.

On the ratio-based measure of adjustment, an asymmetry emerged in Study 2a but did not emerge in Study 2b. In Study 2a, the asymmetry was significant for the pair of sexual assault cases, $b = -0.24$, $SE = 0.02$, $t(583) = -9.71$, $p < .001$; the pair of robbery cases, $b = -0.30$, $SE = 0.04$, $t(583) = -6.68$, $p < .001$; and the pair of aggravated assault cases, $b = -0.21$, $SE = 0.03$, $t(583) = -6.78$, $p < .001$. In Study 2b, however, no asymmetry emerged for any of the three pairs: sexual assault, $b = -1.00$, $SE = 0.82$, $t(582) = -1.21$, $p = .226$; robbery, $b = -0.27$, $SE = 0.21$, $t(582) = -1.28$, $p = .203$; or aggravated assault, $b = 0.12$, $SE = 0.35$, $t(582) = 0.34$, $p = .736$.

When examining whether participants differentiated between cases at all, an asymmetry emerged for all three pairs of transgressions in both studies. In Study 2a, participants who started with the more severe case assigned equal punishment more often than those who started with the

less severe case, and this effect held for the pair of sexual assault cases, $b = 1.83$, $OR = 6.21$, $SE = 0.18$, $z = -9.90$, $p < .001$; the pair of robbery cases, $b = 2.09$, $OR = 8.08$, $SE = 0.19$, $z = 10.85$, $p < .001$; and the pair of aggravated assault cases, $b = 1.26$, $OR = 3.54$, $SE = 0.18$, $z = 7.13$, $p < .001$. Likewise, in Study 2b, this asymmetry emerged for the pair of sexual assault cases, $b = 2.06$, $OR = 4.07$, $SE = 0.20$, $z = 10.57$, $p < .001$; the pair of robbery cases, $b = 0.93$, $OR = 2.54$, $SE = 0.23$, $z = 4.02$, $p < .001$; and the pair of aggravated assault cases, $b = 0.94$, $OR = 2.57$, $SE = 0.20$, $z = 4.67$, $p < .001$.

Punishment Ratings

Table S7 shows the scale-based punishment ratings assigned to each individual case and the amount and frequency of adjustment between cases for transgression type.

In both studies, we found evidence of an order-based asymmetry in adjustment between cases on the scale-based punishment measure. In Study 2a, this effect was significant for the pair of sexual assault cases, $b = -0.16$, $SE = 0.04$, $t(583) = -4.43$, $p < .001$; the pair of robbery cases, $b = -0.18$, $SE = 0.06$, $t(583) = -2.87$, $p = .004$; and the pair of aggravated assault cases, $b = -0.23$, $SE = 0.05$, $t(583) = -4.85$, $p < .001$. In Study 2b, the effect was significant for the pair of sexual assault cases, $b = -0.73$, $SE = 0.07$, $t(582) = -9.78$, $p < .001$; and the pair of robberies, $b = -0.24$, $SE = 0.11$, $t(582) = -2.14$, $p = .033$. However, it was non-significant for the aggravated assault cases, $b = -0.13$, $SE = 0.09$, $t(582) = -1.50$, $p = .134$.

We observed a similar pattern in the frequency with which participants differentiated between cases at all. Participants in Study 2a assigned equal punishment to both cases more often when starting with the more severe case than when starting with the less severe case, and this effect emerged for the pair of sexual assault cases, $b = 0.87$, $OR = 2.38$, $SE = 0.20$, $z = 4.36$, $p < .001$; the pair of robbery cases, $b = 0.79$, $OR = 2.19$, $SE = 0.17$, $z = 4.57$, $p < .001$; and the pair

of aggravated assault cases, $b = 0.59$, $OR = 1.80$, $SE = 0.17$, $z = 3.47$, $p < .001$. In Study 2b, this pattern emerged only for the pair of sexual assault cases, $b = 1.25$, $OR = 3.49$, $SE = 0.17$, $z = 7.18$, $p < .001$. It was marginally significant for the aggravated assault cases, $b = 0.33$, $OR = 1.38$, $SE = 0.18$, $z = 1.86$, $p = .063$; and non-significant for the robbery cases, $b = 0.29$, $OR = 1.33$, $SE = 0.21$, $z = 1.34$, $p = .181$.

Table S7
Punishment of Each Case and Adjustment Between Cases in Studies 2a–2b

		Sexual Assault		Robbery		Aggravated Assault	
		Less Severe First	More Severe First	Less Severe First	More Severe First	Less Severe First	More Severe First
Study 2a	Less Severe Case	9.65 (1.33)	9.77 (1.44)	6.88 (1.81)	6.63 (1.96)	8.84 (1.42)	8.84 (1.63)
	More Severe Case	9.85 (1.21)	9.81 (1.42)	7.44 (1.79)	7.00** (1.89)	9.21 (1.36)	8.98* (1.6)
	Absolute Adjustment	0.20 (0.50)	0.04*** (0.36)	0.56 (0.79)	0.37*** (0.75)	0.37 (0.66)	0.14*** (0.48)
	Frequency of Non-Adjustment	67.1%	82.9%***	31.2%	49.8%***	51.7%	65.9%***
Study 2b	Less Severe Case	9.27 (1.64)	9.37 (1.78)	6.52 (2.05)	6.40 (2.25)	8.37 (1.94)	8.51 (1.89)
	More Severe Case	10.21 (1.26)	9.58*** (1.69)	8.14 (1.81)	7.78* (2.01)	9.30 (1.53)	9.31 (1.60)
	Absolute Adjustment	0.94 (1.03)	0.21*** (0.77)	1.62 (1.34)	1.38* (1.39)	0.93 (1.04)	0.80 (1.09)
	Frequency of Non-Adjustment	38.5%	68.6%***	16.5%	20.8%	30.6%	37.9%†

Note: Standard deviations are given in parentheses. Indications of statistical significance are based on linear regressions with the amount participants adjusted scale-based punishment between the two cases (more severe minus less severe) predicted by order of evaluation (-0.5 = less-severe-first, +0.5 = more-severe-first). † $p < .10$, * $p < .05$, ** $p < .01$, *** $p < .001$

Interpretation of Discrepancies Between Studies 2a and 2b

There are several possible explanations for the discrepancies between the transgression-specific patterns in Studies 2a and 2b. First, it is possible that the asymmetry is stronger for pairs of quantitatively different harms than for pairs of qualitatively different harms. However, note that we observed strong asymmetries in the pair of qualitatively different sexual assault cases and the pair of qualitatively different aggravated assault cases, which casts doubt on this explanation.

Second, it is possible that the asymmetry is stronger for pairs of transgressions that are perceived as more similar to each other, perhaps because people are more likely to make spontaneous comparisons between them. Our reluctance to downplay account should only apply when people are making judgments that they construe as comparative. If people do not engage in comparison, or fail to notice similarities between cases, they should not show an asymmetry. Consistent with this possibility, we observed the strongest asymmetries for pairs of transgressions in which the action itself remains the same across cases and severity is determined by other factors (e.g., number of victims, age of victim). By contrast, the only pair for which we observed no asymmetry was the pair of qualitatively different robbery cases in Study 2b, which involved different actions altogether (i.e., raising a fist at a cashier vs. pointing a gun at a cashier). However, given that we find some evidence of asymmetric adjustment *between* different pairs of transgressions, we think it is unlikely that similarity between cases explains why we observed no asymmetry for the robbery cases in Study 2b.

A third possibility is that the specific form of robbery that we chose for the less severe robbery case in Study 2b is simply not very aversive to downplay. According to our theory, people should be more reluctant to downplay—and thus adjust condemnation more asymmetrically—when comparing transgressions for which they see condemnation as a stronger

signal of good moral character. If the less severe form of robbery used in Study 2b (i.e., raising a fist at a cashier and threatening to hurt her) was not perceived as a strong signal of morality, then this could explain why participants did not show any asymmetry for this pair in particular.

Study 3

Punishment

Participants refrained from differentiating punishment (i.e., assigned equal punishment to both cases within a pair) more frequently when asked which case deserved *less* (vs. *more*) punishment was significant within every pair of transgressions (Table S8).

Moral Wrongness

The effect of comparison frame on refraining from differentiating in moral wrongness (i.e., indicating that both cases within a pair were equally wrong) went in the same direction as the overall effect for every pair of transgressions. It was significant for every pair of transgressions except for robbery (Table S9).

Table S8
Punishment Responses for Each Pair of Transgressions by Frame in Study 3

	Aggravated Assault		Kidnapping		Manslaughter		Robbery		Sexual Assault	
Response	*More* Frame	*Less* Frame	*More* Frame	*Less* Frame	*More* Frame	*Less* Frame	*More* Frame	*Less* Frame	*More* Frame	*Less* Frame
Equal Punishment	36.4%	64.9%***	44.1%	78.0%***	38.1%	63.2%***	20.5%	30.4%**	35.0%	63.2%***
More Punishment for More Severe Case	62.3%	33.8%***	54.2%	20.3%***	60.3%	36.2%***	77.8%	67.2%**	64.7%	35.5%***
More Punishment for Less Severe Case	1.4%	1.4%	1.7%	1.7%	1.7%	0.7%	1.7%	2.4%	0.3%	1.4%

Table S9
Moral Wrongness Responses for Each Pair of Transgressions by Frame in Study 3

	Aggravated Assault		Kidnapping		Manslaughter		Robbery		Sexual Assault	
Response	*More* Frame	*Less* Frame	*More* Frame	*Less* Frame	*More* Frame	*Less* Frame	*More* Frame	*Less* Frame	*More* Frame	*Less* Frame
Equally Wrong	79.5%	87.5%**	85.5%	92.6%**	90.2%	93.2%	72.1%	78.7%†	58.3%	74.7%***
More Severe Case is More Wrong	20.5%	11.5%**	13.8%	6.1%**	9.8%	5.7%†	27.3%	19.3%*	41.8%	25.0%***
Less Severe Case is More Wrong	0.0%	1.0%†	0.7%	1.4%	0.0%	1.0%†	0.7%	2.0%	0.0%	0.3%

Note: These tables show the proportion of participants choosing each response option. Indications of statistical significance are based on linear regressions comparing the choice of each option in the "more" (vs. "less") condition. † $p < .10$, * $p < .05$, ** $p < .01$, *** $p < .001$

Tests of Asymmetric Adjustment Between Transgression Types

Because participants in Studies 2a and 2b evaluated three different pairs of transgressions in random order, we were also able to test for asymmetric adjustment *between* different types of transgressions. These analyses were unplanned and are purely exploratory.

For each dependent variable and for each pair of transgressions, we began by averaging a participant's responses for the less severe and more severe case. We then calculated three difference scores between a participant's average condemnation of one category and their average condemnation of each other category (more severe minus less severe). The ordering of each category's severity was based on its ranking on moral conviction and outrage: Sexual assault was consistently judged as most severe, followed by aggravated assault, followed by robbery. We used the (random) order in which participants evaluated each pair to assign each observation to the less-severe-category-first or the more-severe-category-first condition. For instance, a participant who evaluated aggravated assault first, sexual assault second, and robbery third would be assigned to the less-severe-category-first condition for the sexual assault versus aggravated assault comparison, and to the more-severe-category-first condition for the sexual assault versus robbery comparison. We excluded data for the two categories that did not appear adjacent to each other (i.e., the categories that a participant evaluated first and third), given that participants did not directly adjust between these two categories. Each participant thus provided two observations for each dependent variable.

Differentiating Punishment Length Across Categories (Open-Ended)

For this analysis, we applied the same exclusion rule that was applied to our primary analyses of punishment length adjustment. We conducted a linear regression with adjustment of punishment length predicted by category order (-0.5 = less-severe-category-first, +0.5 = more-

severe-category-first), with fixed effects for each pair of categories and with standard errors clustered by participant. In both studies, overall, we observed a between-category asymmetry similar to the one we observed within transgression categories. In Study 2a, participants who started with the less severe category adjusted punishment length by an average of 19.46 (SD = 23.55) years between categories, while those who started with the more severe category adjusted by an average of 14.47 (SD = 22.91) years, b = -4.76, clustered SE = 0.91, t = -5.25, p < .001. In Study 2b, participants who started with the less severe category adjusted punishment length by an average of 13.82 (SD = 20.04) years between categories, while those who started with the more severe category adjusted by an average of 10.21 (SD = 18.66) years, b = -3.60, clustered SE = 0.79, t = -4.53, p < .001. Table S9 shows the average adjustment amounts between each pair of transgression types.

Table S9
Adjustment of Punishment Length Between Pairs of Transgressions in Studies 2a–2b

	Robbery (Less Severe) vs. Aggravated Assault (More Severe)		Robbery (Less Severe) vs. Sexual Assault (More Severe)		Aggravated Assault (Less Severe) vs. Sexual Assault (More Severe)	
	Less Severe Category First	More Severe Category First	Less Severe Category First	More Severe Category First	Less Severe Category First	More Severe Category First
Study 2a	13.69 (15.87)	13.22 (16.98)	28.80 (27.17)	25.19 (26.68)	15.91 (23.38)	5.56*** (20.36)
Study 2b	10.69 (15.54)	8.84 (14.24)	17.80 (22.41)	17.01 (22.25)	13.04 (20.99)	5.07*** (16.56)

Note: Standard deviations are given in parentheses. Punishment length for a given category was the average punishment length assigned to the less severe case and the more severe case within that category. Indications of statistical significance are based on linear regressions with the amount participants adjusted the length of punishment between two categories (more severe minus less severe) predicted by order of evaluation (-0.5 = less-severe-category-first, +0.5 = more-severe-category-first). The ranking that we used for the relative severity of each category was: sexual assault, aggravated assault, robbery. † p < .10, * p < .05, ** p < .01, *** p < .001

Differentiating Punishment Across Categories (Scale-Based)

We observed similar patterns when testing for asymmetric adjustment between categories using the scale-based punishment measure. In Study 2a, participants who started with the less severe category adjusted punishment by an average of 2.03 (*SD* = 1.73) points on the 11-point scale between categories, while those who started with the more severe category adjusted by an average of 1.51 (*SD* = 1.82) points, $b = -0.52$, clustered $SE = 0.07$, $t = -7.80$, $p < .001$. In Study 2b, participants who started with the less severe category adjusted by an average of 1.83 (*SD* = 1.63) scale points, while those who started with the more severe category adjusted by an average of 1.33 (*SD* = 1.74) points, $b = -0.49$, clustered $SE = 0.07$, $t = -7.07$, $p < .001$. Table S10 shows the average adjustment amounts between each pair of transgression types.

Table S10
Adjustment of Scale-Based Punishment Between Pairs of Transgressions in Studies 2a–2b

	Robbery (Less Severe) vs. Aggravated Assault (More Severe)		Robbery (Less Severe) vs. Sexual Assault (More Severe)		Aggravated Assault (Less Severe) vs. Sexual Assault (More Severe)	
	Less Severe Category First	More Severe Category First	Less Severe Category First	More Severe Category First	Less Severe Category First	More Severe Category First
Study 2a	1.94 (1.38)	1.68 (1.51)	3.09 (1.84)	2.56 (1.99)	1.08 (1.27)	0.29 (1.09)
Study 2b	1.68 (1.24)	1.51 (1.60)	2.78 (1.82)	2.13*** (1.88)	1.05 (1.26)	0.43*** (1.24)

Note: Standard deviations are given in parentheses. Punishment length for a given category was the average punishment length assigned to the less severe case and the more severe case within that category. Indications of statistical significance are based on linear regressions with the amount participants adjusted punishment between two categories (more severe minus less severe) predicted by order of evaluation (-0.5 = less-severe-category-first, +0.5 = more-severe-category-first). The ranking that we used for the relative severity of each category was: sexual assault, aggravated assault, robbery. † p < .10, * p < .05, ** p < .01, *** p < .001

Tests of Asymmetric Adjustment of Moral Outrage

Studies 2a–2b

Adjustment of Moral Outrage Between Cases

In Studies 2a–2b, we included an exploratory measure of moral outrage for each case. We specified in our preregistration that we would test for an order-based asymmetry in the amount that participants adjusted moral outrage between cases. In both studies, we observed an order-based asymmetry in adjustment of moral outrage similar to those we observed in punishment. In Study 2a, participants who started with the less severe case adjusted outrage upward to a greater extent ($M = 0.22$, $SD = 0.72$) than those who started with the more severe case adjusted downward ($M = 0.15$, $SD = 0.62$), $b = -0.07$, clustered $SE = 0.03$, $t = -2.04$, $p = .042$. Similarly, in Study 2b, participants who started with the less severe case adjusted upward to a greater extent ($M = 0.91$, $SD = 1.29$) than those who started with the more severe case adjusted downward ($M = 0.73$, $SD = 1.20$), $b = -0.18$, clustered $SE = 0.06$, $t = -2.92$, $p = .003$.

When examining the amount of outrage expressed toward each case individually, rather than adjustment *between* cases, we found no order effects on the less severe case in either study, $|t|$s < 1. There was also no effect of order on outrage toward the more severe transgression in Study 2a, $t = -1.45$, $p = .145$. However, in Study 2b, participants who started with the more severe case expressed significantly less outrage toward the more severe case than those who started with the less severe case, $b = -0.29$, clustered $SE = 0.11$, $t = -2.57$, $p = .010$. Table S11 shows moral outrage judgments for each case in each study.

Table S11
Mean Moral Outrage for Each Case in Studies 2a–2b

		Sexual Assault		Robbery		Aggravated Assault	
		Less Severe First	More Severe First	Less Severe First	More Severe First	Less Severe First	More Severe First
Study 2a	Less Severe Case	10.49 (1.00)	10.40 (1.10)	6.41 (2.39)	6.28 (2.54)	9.57 (1.59)	9.54 (1.61)
	More Severe Case	10.52 (0.98)	10.47 (1.01)	6.76 (2.38)	6.49 (2.53)	9.83 (1.44)	9.71 (1.51)
Study 2b	Less Severe Case	10.13 (1.36)	9.95 (1.75)	7.00 (2.41)	7.00 (2.60)	9.34 (1.69)	9.19 (2.02)
	More Severe Case	10.65 (0.83)	10.24*** (1.46)	8.59 (2.02)	8.26† (2.36)	9.95 (1.37)	9.82 (1.76)

Note: Standard deviations are given in parentheses. Indications of statistical significance are based on linear regressions comparing outrage toward a given case in the more-severe-first condition versus the less-severe-first condition. † $p < .10$, * $p < .05$, ** $p < .01$, *** $p < .001$

Supplemental Study 1

Adjustment of Moral Outrage Between Cases

In Supplemental Study 1, unlike in Studies 2a–2b, we found no evidence of an order-based asymmetry in moral outrage: Participants who started with the less severe case adjusted moral outrage no more or less (M = 1.05, SD = 1.36) than those who started with the more severe case (M = 1.01, SD = 1.39), b = 0.04, clustered SE = 0.07, t = 0.61, p = .539. Order did not have a significant effect on moral outrage toward the more severe transgression individually, t = -1.02, p = .309, nor toward the less severe transgression individually, $|t| < 1$.

Order Effects on Less Severe and More Severe Cases Individually

Studies 2a–2b

Less Severe Case

In both studies, participants assigned significantly longer punishments to the less severe case when they evaluated it after the more severe case than when they evaluated it first. In Study 2a, those who started with the less severe case assigned it an average of 20.70 (SD = 21.19) years of punishment, while those who started with the more severe case assigned it 24.47 (SD = 24.40) years, b = 3.62, clustered SE = 1.34, t = 2.71, p = .007. In Study 2b, those who started with the less severe case assigned it an average of 11.34 (SD = 11.56) years of punishment, while those who started with the more severe case assigned it 14.33 (SD = 15.17) years, b = 3.01, clustered SE = 0.80, t = 3.76, p < .001. Table 1 in the main text shows order effects on punishment length for each individual transgression. Overall, order did not significantly impact scale-based punishment (Table S12) for the less severe case in either study, $|t|$s < 1.

More Severe Case

In contrast, order of evaluation did not have a significant effect on the length of punishment assigned to the more severe case in either study. In Study 2a, participants who started with the less severe case assigned it an average of 27.23 (SD = 25.49) years of punishment, while those who started with the more severe case assigned it an average of 26.80 (SD = 25.23) years, b = 0.59, clustered SE = 1.52, t = 0.39, p = .699. In Study 2b, participants who started with the less severe case assigned it an average of 18.79 (SD = 15.97) years of prison time, while those who started with the more severe case assigned it an average of 18.45 (SD = 16.26) years, b = -0.32, clustered SE = 1.04, t = -0.31, p = .757.

The opposite pattern emerged in scale-based punishment: Though punishment of the less severe transgression was not influenced by order, punishment of the more severe transgression was significantly greater when it was evaluated first than when it was evaluated second in Study 2a, $b = -0.23$, clustered $SE = 0.10$, $t = -2.27$, $p = .023$, and in Study 2b, $b = -0.33$, clustered $SE = 0.11$, $t = -2.86$, $p = .004$. Table S12 shows scale-based punishments for each case.

Table S12
Mean Punishment for Each Case in Studies 2a–2b

		Sexual Assault		Robbery		Aggravated Assault	
		Less Severe First	More Severe First	Less Severe First	More Severe First	Less Severe First	More Severe First
Study 2a	Less Severe Case	9.65 (1.33)	9.77 (1.44)	6.88 (1.81)	6.63 (1.96)	8.84 (1.42)	8.84 (1.63)
	More Severe Case	9.85 (1.21)	9.81 (1.42)	7.44 (1.79)	7.00** (1.89)	9.21 (1.36)	8.98* (1.6)
Study 2b	Less Severe Case	9.27 (1.64)	9.37 (1.78)	6.52 (2.05)	6.40 (2.25)	8.37 (1.94)	8.51 (1.89)
	More Severe Case	10.21 (1.26)	9.58*** (1.69)	8.14 (1.81)	7.78* (2.01)	9.30 (1.53)	9.31 (1.60)

Note: Standard deviations are given in parentheses. Indications of statistical significance are based on linear regressions comparing punishment of a given case in the more-severe-first condition versus the less-severe-first condition. † $p < .10$, * $p < .05$, ** $p < .01$, *** $p < .001$

Moderation Analyses for Studies 2a–2b and Supplemental Study 1

We conducted a series of exploratory moderation analyses to probe whether the order-based asymmetry in Studies 2a–2b and Supplemental Study 1 was moderated by the degree of moral conviction and/or moral outrage evoked by a given transgression type. Table S13 summarizes the evidence for moderation for each dependent measure in each study.

Moral Conviction

At the end of both studies, we measured participants' moral conviction about each transgression category (i.e., sexual assault, robbery, and aggravated assault). Moral conviction is a meta-cognition that reflects the extent to which an attitude is rooted in one's sense of right and wrong (Skitka & Morgan, 2014). We considered the possibility that the strength of people's moral conviction about a pair of transgressions might determine the extent to which they adjust condemnation asymmetrically between those transgressions. We tested for moderation both at the participant level (given that different individuals might have stronger moral convictions about an issue than others) and at the level of the transgression type (given that the size of the asymmetry might be driven more by variation in the strength of moral attitudes toward a transgression type *in general* than by the strength of the individual's attitudes).

As a Participant-Level Moderator

First, we explored whether *individual differences* in moral conviction moderated the size of the order-based asymmetry in adjustment of punishment. These analyses test whether participants who held stronger moral convictions about a type of transgression adjusted more asymmetrically when sequentially evaluating two specific transgressions of this type.

Prison Sentences. We conducted a linear regression with punishment length adjustment predicted by (1) order of evaluation (-0.5 = less-severe-first, 0.5 = more-severe-first), (2) moral

conviction (mean-centered), (3) a variable for the pair of aggravated assault cases (-0.25 = robbery or sexual assault, +0.5 = aggravated assault), (4) a variable for the pair of sexual assault cases (-0.25 = robbery or aggravated assault, +0.5 = sexual assault), and (5-9) all two-way interactions between these variables. Standard errors were clustered at the participant level. We were primarily interested in the order X moral conviction interaction: A negative sign would indicate that participants who held stronger moral conviction about a type of transgression adjusted more asymmetrically between two specific cases of this transgression type. The interaction term was negative but non-significant in Study 2a, $b = -0.48$, clustered $SE = 0.55$, $t = -0.87$, $p = .385$; Study 2b, $b = -0.54$, clustered $SE = 0.59$, $t = -0.90$, $p = .366$; and Supplemental Study 1, $b = -0.72$, clustered $SE = 0.44$, $t = -1.62$, $p = .106$.

Punishment Ratings. We observed directionally consistent (albeit non-significant) interaction effects when we replaced punishment length adjustment with adjustment on the scale-based punishment measure. The interaction between moral conviction and order of evaluation was not significant in Study 2a, $b = -0.06$, clustered $SE = 0.04$, $t = -1.60$, $p = .109$; Study 2b, $b = -0.08$, clustered $SE = 0.07$, $t = -1.20$, $p = .230$; or Supplemental Study 1, $b = -0.08$, clustered $SE = 0.07$, $t = -1.01$, $p = .310$.

As a Transgression-Type-Level Moderator

Second, we examined whether the *average* moral conviction about each category of transgressions moderated the extent to which participants adjusted punishment in each direction. To do so, we first averaged moral conviction about a given category across all participants. In every study, participants held the strongest moral convictions about sexual assault ($M_{2a} = 4.68$, $SD = 0.71$; $M_{2b} = 4.50$, $SD = 0.87$; $M_{SS1} = 4.47$, $SD = 0.85$), followed by aggravated assault (M_{2a}

= 4.23, *SD* = 0.82; M_{2b} = 4.16, *SD* = 0.93; M_{SS1} = 4.11, *SD* = 0.91), and robbery (M_{2a} = 3.54, *SD* = 0.94; M_{2b} = 3.93, *SD* = 0.99; M_{SS1} = 3.66, *SD* = 1.05).

Prison Sentences. We conducted a regression with adjustment of punishment length predicted by (1) order (-0.5 = less-severe-first, 0.5 = more-severe-first), (2) average category-level moral conviction, and (3) the two-way interaction between these variables, with standard errors clustered by participant. The interaction between average moral conviction and order of evaluation was significant in Study 2a, b = -1.82, clustered *SE* = 0.66, t = -2.76, p = .006; Study 2b, b = -13.09, clustered *SE* = 1.70, t = -7.72, p < .001; and Supplemental Study 1, b = -7.85, clustered *SE* = 0.90, t = -8.74, p < .001. This reflects that the types of transgressions that were associated with stronger moral conviction (in general) produced a larger order-based asymmetry.

Punishment Ratings. In the model predicting adjustment on the scale-based punishment measure, the interaction between average category-level moral conviction and order of evaluation was non-significant in Study 2a, b = 0.01, clustered *SE* = 0.06, t = 0.20, p = .843. However, the interaction was negative and significant in Study 2b, b = -0.93, clustered *SE* = 0.23, t = -4.10, p < .001, and Supplemental Study 1, b = -0.67, clustered *SE* = 0.16, t = -4.23, p < .001, in line with the pattern of results that emerged on the open-ended measure. In other words, the asymmetry was no more pronounced for transgression types associated with stronger moral conviction.

Moral Outrage

We also repeated the same procedure with moral outrage, which was measured for each individual transgression within a pair rather than for the broader category of transgression. We created a moral outrage composite for each participant and for each pair of transgressions by averaging moral outrage responses across the less severe case and the more severe case (Study

2a: $r = .96$; Study 2b: $r = .85$; Supplemental Study 1: $r = .87$). Moral outrage had a moderate positive correlation with moral conviction (Study 2a: $r = .55$; Study 2b: $r = .42$; Supplemental Study 1: $r = .50$). This suggests that the strength of participants' moral conviction about a transgression category did not map perfectly onto the degree of outrage they felt toward two specific cases of that category. Therefore, we separately tested whether transgressions that evoked greater outrage produced a larger order-based asymmetry. As with moral conviction, we tested for moderation both at the participant level and at the transgression-type level.

As a Participant-Level Moderator

Prison Sentences. We conducted a linear regression with punishment length adjustment predicted by (1) order of evaluation (-0.5 = less-severe-first, 0.5 = more-severe-first), (2) average moral outrage toward the pair of transgressions (mean-centered), (3) a variable for the pair of aggravated assault cases (-0.25 = robbery or sexual assault, +0.5 = aggravated assault), (4) a variable for the pair of sexual assault cases (-0.25 = robbery or aggravated assault, +0.5 = sexual assault), and (5-9) all two-way interactions between these variables, with standard errors clustered by participant. The interaction between moral outrage and order of evaluation was significant in Study 2a, $b = -0.48$, clustered $SE = 0.21$, $t = -2.31$, $p = .021$; Study 2b, $b = -0.73$, clustered $SE = 0.24$, $t = -3.04$, $p = .002$; and Supplemental Study 1, $b = -0.49$, clustered $SE = 0.17$, $t = -2.93$, $p = .003$. This means that participants who felt more outraged toward a pair of transgressions adjusted punishment lengths between those transgressions more asymmetrically.

Punishment Ratings. We observed a similar pattern when examining adjustment of scale-based punishment in Study 2a: The interaction between moral outrage and order of evaluation was significant, $b = -0.05$, clustered $SE = 0.02$, $t = -2.41$, $p = .016$. This interaction

went in the same direction but was not significant in Study 2b, $b = 0.03$, clustered $SE = 0.04$, $t = 0.90$, $p = .370$, and Supplemental Study 1, $b = -0.04$, clustered $SE = 0.04$, $t = -1.03$, $p = .302$.

As a Transgression-Type-Level Moderator

We also examined whether the *average* moral outrage participants expressed toward each pair of transgressions moderated the size of the asymmetry. We first averaged the mean moral outrage for a given pair of transgressions across all participants. In all three studies, participants expressed the most outrage toward the pair of sexual assault cases ($M_{2a} = 10.47$, $SD = 1.01$; $M_{2b} = 10.24$, $SD = 1.32$; $M_{SS1} = 10.08$, $SD = 1.33$), followed by aggravated assault ($M_{2a} = 9.66$, $SD = 1.50$; $M_{2b} = 9.57$, $SD = 1.65$; $M_{SS1} = 8.98$, $SD = 1.85$), and robbery ($M_{2a} = 6.48$, $SD = 2.42$; $M_{2b} = 7.71$, $SD = 2.23$; $M_{SS1} = 6.51$, $SD = 2.52$). Participants did not express any more or less outrage overall (i.e., across all transgression pairs) as a function of order in Study 2a, $t = 1.11$, $p = .267$, or Supplemental Study 1, $t = .81$, $p = .416$. However, in Study 2b, those who started with the less severe case within each pair expressed marginally greater outrage than those who started with the more severe case, $b = 0.20$, clustered $SE = 0.12$, $t = 1.67$, $p = .096$.

Prison Sentences. We regressed adjustment of punishment length predicted by (1) order of evaluation (-0.5 = less-severe-first, 0.5 = more-severe-first), (2) average transgression-type-level moral outrage, and (3) the two-way interaction between these variables, with standard errors clustered by participant. The interaction between average moral outrage and order of evaluation was positive and significant in Study 2a, $b = -0.45$, clustered $SE = 0.18$, $t = -2.57$, $p = .010$; Study 2b, $b = -2.25$, clustered $SE = 0.35$, $t = -6.33$, $p < .001$; and Supplemental Study 1, $b = -1.55$, clustered $SE = 0.19$, $t = -8.10$, $p < .001$. Similar to the patterns we observed in category-level moral conviction, types of transgressions that evoked greater outrage (in general) produced a larger order-based asymmetry.

Punishment Ratings. In the model predicting adjustment on the scale-based punishment measure, the interaction between average transgression-type-level moral outrage and order of evaluation was non-significant in Study 2a, $b = -0.00$, clustered $SE = 0.02$, $t = -0.01$, $p = .994$. However, it was significant in Study 2b, $b = -0.14$, clustered $SE = 0.05$, $t = -2.62$, $p = .009$, and in Supplemental Study 1, $b = -0.13$, clustered $SE = 0.04$, $t = -3.52$, $p < .001$. This suggests that, consistent with the open-ended punishment length measure, the order-based asymmetry in scale-based punishment may have been especially pronounced for pairs of transgressions that (in general) evoked greater outrage.

Table S13

Moral Conviction and Moral Outrage as Moderators of Order-Based Asymmetry in Studies 2a–2b and Supplemental Study 1

Study	***Dependent Variable***	***Order X Moral Conviction Interaction (Participant-Level)***	***Order X Moral Conviction Interaction (Transgression-Type-Level)***	***Order X Moral Outrage Interaction (Participant-Level)***	***Order X Moral Outrage Interaction (Transgression-Type-Level)***
**2a**	Differentiating Punishment Length (Open-Ended)	$b = -0.48$, $SE = 0.55$, $t = -0.87$, $p = .385$	**$b = -1.82$, $SE = 0.66$, $t = -2.76$, $p = .006$**	**$b = -0.48$, $SE = 0.21$, $t = -2.31$, $p = .021$**	**$b = -0.45$, $SE = 0.18$, $t = -2.57$, $p = .010$**
	Differentiating Punishment Length (Ratio-Based)	$b = -0.01$, $SE = 0.03$, $t = -0.37$, $p = .712$	$b = 0.06$, $SE = 0.04$, $t = 1.40$, $p = .161$	$b = 0.02$, $SE = 0.01$, $t = 1.16$, $p = .244$	$b = 0.02$, $SE = 0.01$, $t = 1.54$, $p = .124$
	Refraining from Differentiating Punishment Length (Binary)	**$b = 0.37$, $SE = 0.15$, $z = 2.51$, $p = .012$**	$b = -0.30$, $SE = 0.20$, $z = -1.50$, $p = .134$	$b = 0.03$, $SE = 0.07$, $z = 0.48$, $p = .631$	$b = -0.12$, $SE = 0.06$, $z = -2.14$, $p = .032$
	Differentiating Punishment (Scale-Based)	$b = -0.06$, $SE = 0.04$, $t = -1.60$, $p = .109$	$b = 0.01$, $SE = 0.06$, $t = 0.20$, $p = .843$	**$b = -0.05$, $SE = 0.02$, $t = -2.41$, $p = .016$**	$b = -0.00$, $SE = 0.02$, $t = -0.01$, $p = .994$
	Refraining from Differentiating Punishment (Binary)	$b = 0.09$, $SE = 0.13$, $z = 0.64$, $p = .524$	$b = -0.00$, $SE = 0.21$, $z = -0.03$, $p = .976$	$b = 0.07$, $SE = 0.06$, $z = 1.07$, $p = .287$	$b = -0.02$, $SE = 0.06$, $z = -0.36$, $p = .715$
**2b**	Differentiating Punishment Length (Open-Ended)	$b = -0.54$, $SE = 0.59$, $t = -0.90$, $p = .366$	**$b = -13.09$, $SE = 1.70$, $t = -7.72$, $p < .001$**	**$b = -0.73$, $SE = 0.24$, $t = -3.04$, $p = .002$**	**$b = -2.25$, $SE = 0.35$, $t = -6.33$, $p < .001$**
	Differentiating Punishment Length (Ratio-Based)	$b = 0.96$, $SE = 0.98$, $t = 0.98$, $p = .329$	$b = -1.43$, $SE = 1.56$, $t = -0.92$, $p = .359$	$b = 0.02$, $SE = 0.10$, $t = 0.17$, $p = .863$	$b = -0.18$, $SE = 0.27$, $t = -0.67$, $p = .504$
	Refraining from Differentiating Punishment Length (Binary)	$b = 0.17$, $SE = 0.14$, $z = 1.18$, $p = .239$	**$b = 2.18$, $SE = 0.50$, $z = 4.32$, $p < .001$**	$b = 0.01$, $SE = 0.10$, $z = 0.05$, $p = .957$	**$b = 0.38$, $SE = 0.12$, $z = 3.22$, $p = .001$**
	Differentiating Punishment (Scale-Based)	$b = -0.08$, $SE = 0.07$, $t = -1.20$, $p = .230$	**$b = -0.93$, $SE = 0.23$, $t = -4.10$, $p < .001$**	$b = 0.03$, $SE = 0.04$, $t = 0.90$, $p = .370$	**$b = -0.14$, $SE = 0.05$, $t = -2.62$, $p = .009$**
	Refraining from Differentiating Punishment (Binary)	$b = 0.01$, $SE = 0.17$, $z = 0.04$, $p = .964$	**$b = 1.87$, $SE = 0.43$, $z = 4.41$, $p < .001$**	$b = -0.20$, $SE = 0.11$, $z = -1.93$, $p = .054$	**$b = 0.32$, $SE = 0.10$, $z = 3.10$, $p = .002$**
**Supplemental Study 1**	Differentiating Punishment Length (Open-Ended)	$b = -0.72$, $SE = 0.44$, $t = -1.62$, $p = .106$	**$b = -7.85$, $SE = 0.90$, $t = -8.74$, $p < .001$**	**$b = -0.49$, $SE = 0.17$, $t = -2.93$, $p = .003$**	**$b = -1.55$, $SE = 0.19$, $t = -8.10$, $p < .001$**

	Differentiating Punishment Length (Ratio-Based)	**$b = -0.46$, $SE = 0.17$, $t = -2.68$, $p = .007$**	$b = -0.34$, $SE = 0.31$, $t = -1.13$, $p = .259$	$b = -0.08$, $SE = 0.06$, $t = -1.30$, $p = .193$	$b = -0.06$, $SE = 0.07$, $t = -0.79$, $p = .431$
	Refraining from Differentiating Punishment Length (Binary)	**$b = 0.32$, $SE = 0.18$, $z = 1.81$, $p = .071$**	**$b = 2.26$, $SE = 0.49$, $z = 4.60$, $p < .001$**	**$b = 0.21$, $SE = 0.10$, $z = 2.04$, $p = .042$**	**$b = 0.47$, $SE = 0.11$, $z = 4.15$, $p < .001$**
	Differentiating Punishment (Scale-Based)	$b = -0.08$, $SE = 0.07$, $t = -1.01$, $p = .310$	**$b = -0.67$, $SE = 0.16$, $t = -4.23$, $p < .001$**	$b = -0.04$, $SE = 0.04$, $t = -1.03$, $p = .302$	**$b = -0.13$, $SE = 0.04$, $t = -3.52$, $p < .001$**
	Refraining from Differentiating Punishment (Binary)	$b = 0.01$, $SE = 0.15$, $z = 0.09$, $p = .926$	**$b = 1.80$, $SE = 0.41$, $z = 4.42$, $p < .001$**	**$b = 0.16$, $SE = 0.09$, $z = 1.79$, $p = .073$**	**$b = 0.37$, $SE = 0.10$, $z = 3.89$, $p < .001$**

Note: This table includes tests of the two-way interaction between order of evaluation (-0.5 = less-severe-first, +0.5 = more-severe-first) and moral conviction/outrage (either measured at the participant-category level or averaged across all participants for a given category) as a predictor of differentiating punishment. Each test shown in this table is from a different regression model. Boldface indicates that the order-based asymmetry was significantly larger for transgressions associated with greater moral conviction/outrage.

Additional Analyses for Study 5

We conducted a series of exploratory analyses to test how the size of the asymmetry correlated with each of the three potential moderators in Study 5 (moral signaling relevance, harmfulness, and intentionality) across the 12 transgression types. These analyses test for similar patterns as our preregistered participant-level analyses reported in the main manuscript.

Below, we report correlations between the size of the asymmetry and each potential moderator. In addition to testing for the significance of each correlation, we also test for differences *between* these correlations. Note that because these analyses are conducted at the transgression-type level, and thus are based on only 12 observations each (one per transgression type), they may be underpowered to detect differences between correlations.

Transgression-Level Analyses

Rank-Based Correlations

First, we explored how the ranking of each transgression type in terms of the size of the *more*/*less* asymmetry correlated with its ranking on moral signaling relevance, harmfulness, and intentionality. To do so, we assigned ranks to each transgression type based on the size of the asymmetry for the pair of transgressions of that type in the main study and the average ratings of moral signaling relevance, harmfulness, and intentionality of the transgression type in the pretest, with higher ranks indicating larger values.

As reported in the main manuscript, across the 12 transgression types, the relative size of the *more*/*less* asymmetry was most strongly associated with the transgression type's moral signaling relevance, $r = .881$, $p < .001$, followed by its perceived harmfulness, $r = .783$, $p = .003$, followed by its perceived intentionality, $r = .517$, $p = .085$.

The correlation between the size of the asymmetry and moral signaling relevance correlation did not significantly differ from the correlation with harmfulness, $z = 0.86$, $p = .391$. However, it was significantly greater than the correlation with intentionality, $z = 2.52$, $p = .012$. The harmfulness and intentionality correlations did not differ significantly, $z = 1.04$, $p = .299$.

Raw Correlations

Second, we explored how the absolute magnitude of the *more*/*less* asymmetry correlated with a transgression type's average moral signaling relevance, harmfulness, and intentionality. To do so, we simply used the raw regression coefficient for the *more*/*less* asymmetry within each pair of transgressions and calculated the average rating of each transgression type's moral signaling relevance, harmfulness, and intentionality in the pretest.

The results aligned closely with the results of the rank-based correlations. Across the 12 transgression types, the size of the *more*/*less* asymmetry was most strongly associated with a transgression type's moral signaling relevance, $r = .886$, $p < .001$, followed by its perceived harmfulness, $r = .740$, $p = .006$, followed by its perceived intentionality, $r = .335$, $p = .287$.

Again, only the difference between the moral signaling relevance and intentionality correlations was significant, $z = 2.67$, $p = .008$. The difference between the moral signaling relevance and harmfulness correlations was not significant, $z = 1.25$, $p = .211$, nor was the difference between the harmfulness and intentionality correlations, $z = 1.16$, $p = .246$.

Deviations from Preregistrations

Study 1: Typo in Dependent Variable Description

In our preregistration for Study 1 (available on ResearchBox), we mistakenly wrote that punishment would be measured on a 0-11 scale: "How much should [X] be punished for his actions?" (0 = No punishment, 11 = The death penalty). However, the second scale endpoint was an error: We intended to (and did) use a 0-10 scale (0 = No punishment, 10 = The death penalty).

Studies 2a–2b and Supplemental Study 1: Coding of Binary Dependent Variable

Our preregistrations for Studies 2a–2b and Supplemental Study 1 stated that we would explore whether participants adjusted punishment at all (0 = did not adjust, 1 = did adjust). We instead decided to report these results in terms of whether participants *refrained from adjusting* (0 = did adjust, 1 = did not adjust) for consistency across studies. Because this is a binary variable, our results do not change depending on which coding scheme is used.

Study 5: Linear vs. Logistic Regression

Our preregistration for Study 5 stated that we would test for the interaction between comparison frame and each potential moderator of the asymmetry using logistic mixed-effects regressions. However, because these models failed to converge, we instead used linear mixed effects regressions. Our preregistered logistic regression models, despite convergence issues, yielded similar results, and we include both sets of models in the code files posted on ResearchBox. For further discussion of when it is appropriate to use linear regression with a binary dependent variable, see Gomila (2021).

References

Gomila, R. (2021). Logistic or linear? Estimating causal effects of experimental treatments on binary outcomes using regression analysis. *Journal of Experimental Psychology: General*, *150*(4), 700.

Skitka, L. J., & Morgan, G. S. (2014). The social and political implications of moral conviction. *Political Psychology*, *35*, 95–110.
